# Spatiotemporal control of engineered bacteria to express interferon-γ by focused ultrasound for tumor immunotherapy

**DOI:** 10.1038/s41467-022-31932-x

**Published:** 2022-08-02

**Authors:** Yuhao Chen, Meng Du, Zhen Yuan, Zhiyi Chen, Fei Yan

**Affiliations:** 1 The First Affiliated Hospital, Medical Imaging Centre, Hengyang Medical School, University of South China, Hengyang, Hunan 421001 China; 2 Institute of Medical Imaging, Hengyang Medical School, University of South China, Hengyang, Hunan 421001 China; 3 Faculty of Health Sciences, Centre for Cognitive and Brain Sciences, University of Macau, Taipa, Macau SAR, China; 4 Center for Cell and Gene Circuit Design, CAS Key Laboratory of Quantitative Engineering Biology, Shenzhen Institute of Synthetic Biology, Shenzhen Institute of Advanced Technology, Chinese Academy of Sciences, Shenzhen, 518055 China

**Keywords:** Protein delivery, Immunization, Cancer therapy

## Abstract

Bacteria-based tumor therapy has recently attracted wide attentions due to its unique capability in targeting tumors and preferentially colonizing the core area of the tumor. Various therapeutic genes are also harbored into these engineering bacteria to enhance their anti-tumor efficacy. However, it is difficult to spatiotemporally control the expression of these inserted genes in the tumor site. Here, we engineer an ultrasound-responsive bacterium (URB) which can induce the expression of exogenous genes in an ultrasound-controllable manner. Owing to the advantage of ultrasound in tissue penetration, an acoustic remote control of bacterial gene expression can be realized by designing a temperature-actuated genetic switch. Cytokine interferon-γ (IFN-γ), an important immune regulatory molecule that plays a significant role in tumor immunotherapy, is used to test the system. Our results show that brief hyperthermia induced by focused ultrasound promotes the expression of IFN-γ gene, improving anti-tumor efficacy of URB in vitro and in vivo. Our study provides an alternative strategy for bacteria-mediated tumor immunotherapy.

## Abstract (web summary)

Several approaches have been recently proposed to engineer bacteria for cancer immunotherapy. Here the authors design an ultrasound-responsive bacterium for the controlled release of IFNy at the tumor site, promoting anti-tumor immune responses in preclinical models.

## Introduction

Bacteria-mediated tumor therapy has recently attracted wide attentions as tumor-targeting drug delivery system or as anti-tumor live agents by themselves because of the characteristics of solid tumors, such as hypoxic, immunosuppressive, and biochemically unique microenvironment^1–3^. Compared to other drug delivery carriers, bacteria-based delivery system has many advantages, including: (1) bacteria could actively target hypoxic, eutrophic, and immunosuppressive tumor microenvironment, breaking through the resistance of tumor blood vessel and the high interstitial pressure and proliferating in the tumor^4,5^. This occurs primarily due to reduced immune surveillance along with the ability of bacteria to grow within the hypoxic and necrotic tumor core. Because bacteria are both inherently present and selectively grow within tumors, they provide a natural platform for the development of programmable therapeutic delivery vehicles^6,7^. (2) Also, some engineered bacteria can carry therapeutic genes such as encoding PD-L1 single chain antibody or TNF-a through inserting them into prokaryotic expression vector, which not only entrusts them with hereditability along with their hosts, but also makes it possible to combine multiple genes for maximize their anti-tumor efficacy^8–11^. (3) More importantly, the introduction of synthetic biology technologies into these engineered bacteria, such as promoter engineering, smart genetic circuits greatly improved their ability to sense and response to disease states of inflammation, infection, and to control bacterium growth and gene expression, largely improving their selective colonization of tumors and convenient opportunity for tumor drug delivery^12–14^.

To date, many techniques have been developed for studying inducible gene expression in bacteria, including chemical induction, physical stimulation and biological methods^15,16^. Conventionally, systemically administered chemical inducers are not specific to a particular anatomical site. The tumor microenvironment with high interstitial fluid pressure makes it difficult for chemical inducers to accumulate to the ideal concentration, while increasing their doses may possibly bring with potential side effects^17–19^. Some biological methods such as the bacterial quorum sensing system can adjust gene expression in concert with their population size, making it possible to initiate therapeutic gene expression only when their population density reaches a threshold level^14,16^. However, it is difficult for the biological methods to precisely model gene expression in a spatiotemporally controllable manner, especially in live animals. Physical induction approaches, such as ray irradiation and light stimulation, provide high spatiotemporal precision. But the former is ionizing which may cause damage to the normal tissues in the irradiation path^20^, and the latter is limited by the poor penetration into the deep-seated tumors^21^. In contrast, ultrasound has many advantages in noninvasiveness, safety and tissue penetration^22–24^. Importantly, thanks to the thermal effects, the beam waves excited by ultrasound can be focused into the deep tissues and precisely and locally elevate the temperature of irradiated region through converting the mechanical energy into heat energy. Thus, it may provide an ideal remote regulation of bacterial gene expression when combining with temperature-based gene control elements^25^. Our previous studies have used ultrasound as a trigger source for drug release in temperature-sensitive liposomes to achieve targeted controlled release of drugs at tumor sites^26^.

The leftward (PL) and rightward (PR) phage lambda promoters are strong and finely temperature-regulated promoter elements, which have been employed for the production of many recombinant proteins and peptides in some widely used prokaryotic expression vectors^27^. Transcription from either promoter or both in tandem would be repressed in prokaryotic cells growing at low temperature (28–30 °C) by the thermolabile cI857 repressor expressed from the same vector, while a temperature shift to 42–45 °C would rapidly result in the inactivation of cI857 repressor, making it possible to transcribe the appropriately inserted genes under the promoters and to overproduce their encoded recombinant proteins. By using of the thermo-regulated expression system, many heterologous recombinant proteins and peptides have been successfully induced and produced, avoiding the use of special media, toxic or expensive chemical inducers^28,29^.

**Fig. 1:**
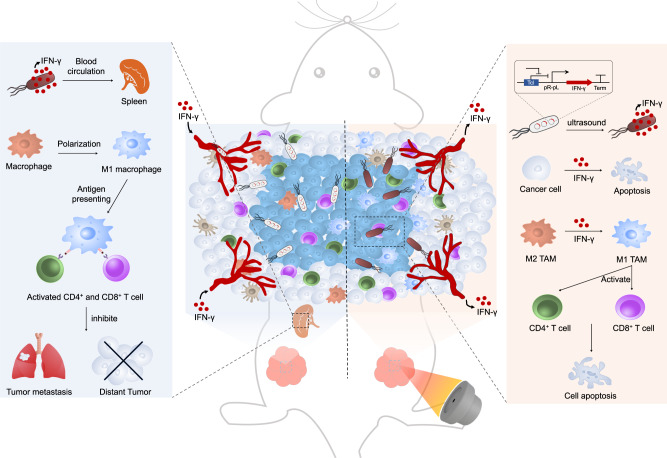
Schematic diagram of URB in controlling IFN-γ expression by focused ultrasound and their mechanisms for cancer immunotherapy. Upon systemic administration of URB that contains IFN-γ gene inserted in the temperature-sensitive genetic circuit, these genetically engineered bacteria would deliver into the tumors due to their tumor-targeting capability. Then, the tumor was irradiated by focused ultrasound to heat these intratumoral bacteria to 42–45°C for inducing the expression of IFN-γ gene. The production and secretion of IFN-γ not only can promote the apoptosis of cancer cells, but also induce the macrophage polarization from M2 to M1 phenotype and the activation of CD4^+^ and CD8^+^ T cells (Right panel).In addition, high levels of IFN-γ can also activate macrophages and T cells in the spleen to inhibit lung metastasis and distant tumor growth through immune memory responses (Left panel).

In this work, we develop an ultrasound-responsive bacterium (URB) which can spatiotemporally control the gene expression by focused ultrasound-induced hyperthermia. In this system, the IFN-γ or mCherry genes are inserted into the multiple clone site under PL and PR tandem promoter and further transformed into the *E.coli* MG1655 that can accumulate in the hypoxic and necrotic areas of tumors (Fig. 1). Upon their delivery into the tumors, focused ultrasound is used to irradiate and heat these bacteria, triggering the expression of IFN-γ gene and activating the anti-tumor immune response. The production and secretion of IFN-γ not only can promote the apoptosis of cancer cells, but also induce the macrophage polarization from M2 to M1 phenotype and the activation of CD4^+^ and CD8^+^ T cells (Fig. 1, right panel). Interestingly, plasma IFN-γ secreting from URB also can polarize the macrophages in murine spleen, enhancing their antigen presenting effects and producing more CD4^+^ and CD8^+^ T cells to inhibit the tumor metastasis and distant tumor growth (Fig. 1, left panel). Thus, a high spatiotemporal controllable gene expression strategy in deep tissues is developed through the remote noninvasive ultrasound in this work.

## Results

### Hyperthermia and ultrasound triggered gene expression of ultrasound-responsive bacterium

**Fig. 2:**
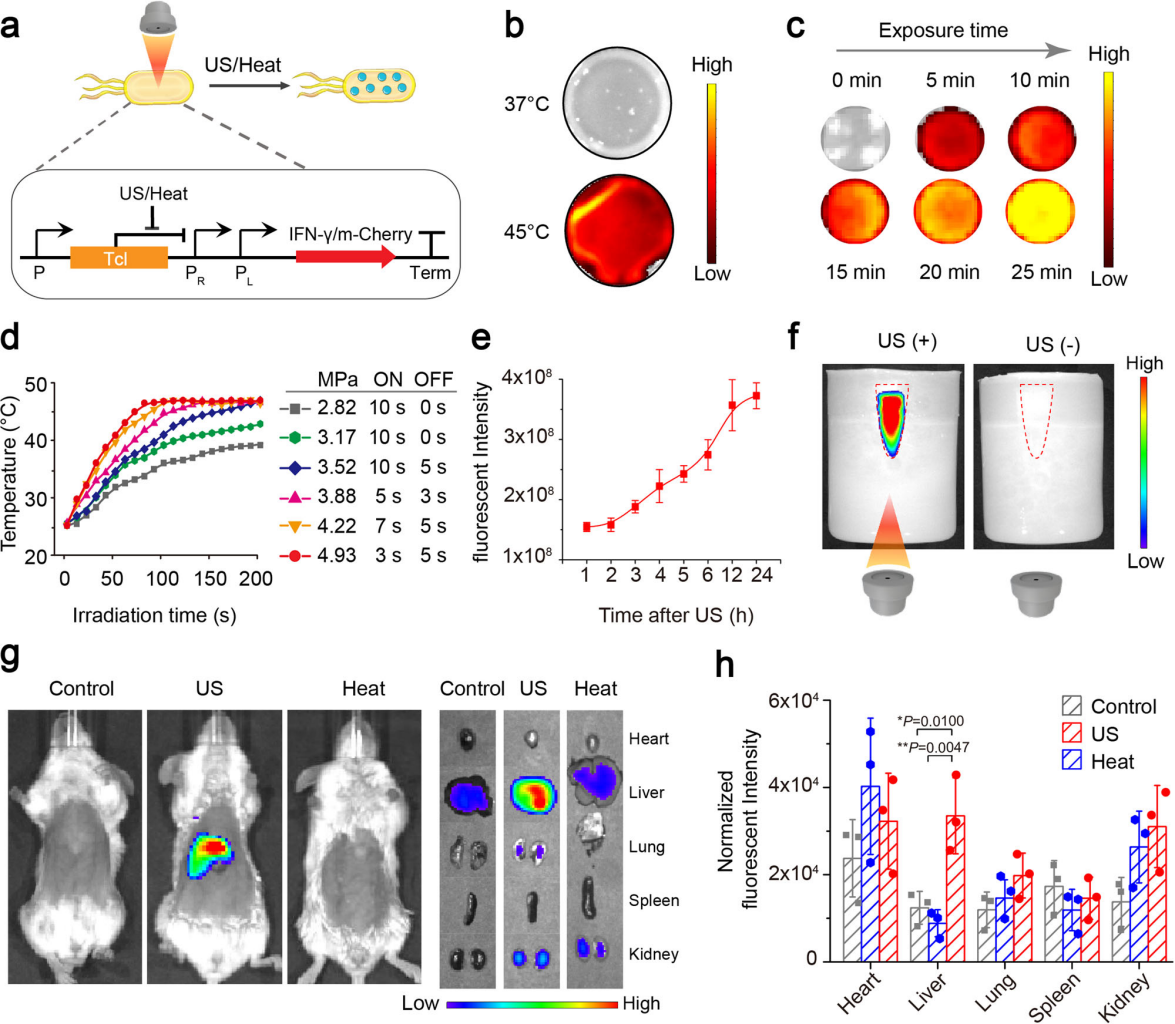
The gene expression of URB trigged by hyperthermia and ultrasound. **a** Mechanism of mCherry/IFN-γ expression based on pBV220 plasmid in the bacteria. **b** Fluorescence image of mCherry protein in URB under different temperature. Images were representative of three experiments. **c** Fluorescence image of mCherry protein in URB under 45 °C with different time. Images were representative of three experiments. **d** Quantification of temperature of bacteria solution irradiated with the different acoustic pressures by focused ultrasound. Experiments were performed three times independently with similar results. **e** Quantification of fluorescence signals of bacteria solution after ultrasound irradiation. *n* = 3 biologically independent samples at each time point. Data were presented as mean ± S.D. **f** Fluorescence image of mCherry protein of URB in the center of the gel phantom under ultrasound irradiation. Images were representative of three experiments. **g** Fluorescence image of mice injected with 1 × 10^7^ CFU of URB under liver-targeted ultrasound irradiation. Images were representative of three experiments. **h** Quantification of fluorescence signals of major organs of mice injected with 1 × 10^7^ CFU of URB under liver-targeted ultrasound irradiation. The fluorescent intensity was normalized by organ size. *n* = 3 biologically independent samples per group. Data were presented as mean ± S.D. Statistical analysis was calculated by using one-way analysis of variance with a Tukey’s test (****P < 0.0001; ***P < 0.001; **P < 0.01; *P < 0.05;). Source data are provided as a Source Data file.

We constructed the prokaryotic expression plasmid harboring temperature-actuated therapeutic circuit which carries murine IFN-γ gene with N-terminal OmpA secretion signal peptide or mCherry fluorescent reporter gene under the pR-pL tandem promoter (Fig. 2a). Then the recombinant plasmid was transformed into *E. coli* MG1655 (a non-pathogenic bacterium) to prepare ultrasound-responsive bacterium (URB). Incubation of the engineered bacteria harboring mCherry gene at 37 °C did not produce any fluorescence signals, showing the suppressed expression of mCherry fluorescent gene at 37 °C. In contrast, strong red fluorescent signals could be observed at 6 h after the bacterium incubation temperature rose to 45 °C, revealing that TcI repression was relieved to initiate the mCherry gene expression (Fig. 2b). Prolonging the bacterial incubation time at 45 °C from 5 min to 25 min increased the fluorescent signal intensities, which confirmed the controllability of thermal logic circuits of URB (Fig. 2c). To develop a noninvasive remote control of bacterial gene circuits by focused ultrasound, we optimized a series of acoustic parameters including acoustic energies, irradiation duration time at ON/OFF state. Results showed that the parameters at 4.93 MPa acoustic pressure, 3 s ON and 5 s OFF periodic irradiation could keep the bacterium solution at 45 °C constant temperature (Fig. 2d). Plating these irradiated URB on the solid LB plates revealed that the activity of bacteria was not affected by water-bath heating or ultrasound irradiation at 45 °C for 30 min (Supplementary Fig. 1). After the ultrasound irradiation, URB could successfully express mCherry fluorescent protein and the fluorescence signal intensity increased gradually along with time, achieving to the peak on day 3, and then gradually fell down until day 7 (Fig. 2e, Supplementary Fig. 2). To elucidate the remote controllability of bacterial gene expression, we inserted a tube filled with bacteria into an agar phantom with or without acoustic exposure for 30 min at the optimal ultrasound parameters. Figure 2f showed the fluorescence signals only appeared in the sample received with ultrasound irradiation, but not in these bacteria that did not receive with ultrasound exposure. In addition to controlling the temperature of the bacterial solution in vitro, the temperature of the local tissue of mice could also be increased to 45 °C by adjusting the appropriate ultrasonic parameters (Supplementary Fig. 3). In vivo animal experiment further revealed that the livers of mice administrated intravenously with URB could emit the strong fluorescence signals after ultrasound irradiation at the above optimized acoustic parameters. By contrast, the livers from the non-irradiated control mice or the incubator-heating mice at 45 °C did just emit weak background fluorescence signals (Fig. 2g, h). These results showed that ultrasound can function as a remote tool to regulate the gene expression of bacteria when they harbored some temperature-based gene control elements in vitro and in vivo.

### In vitro treatment experiment of ultrasound-responsive bacterium-mediated tumor immunotherapy

**Fig. 3:**
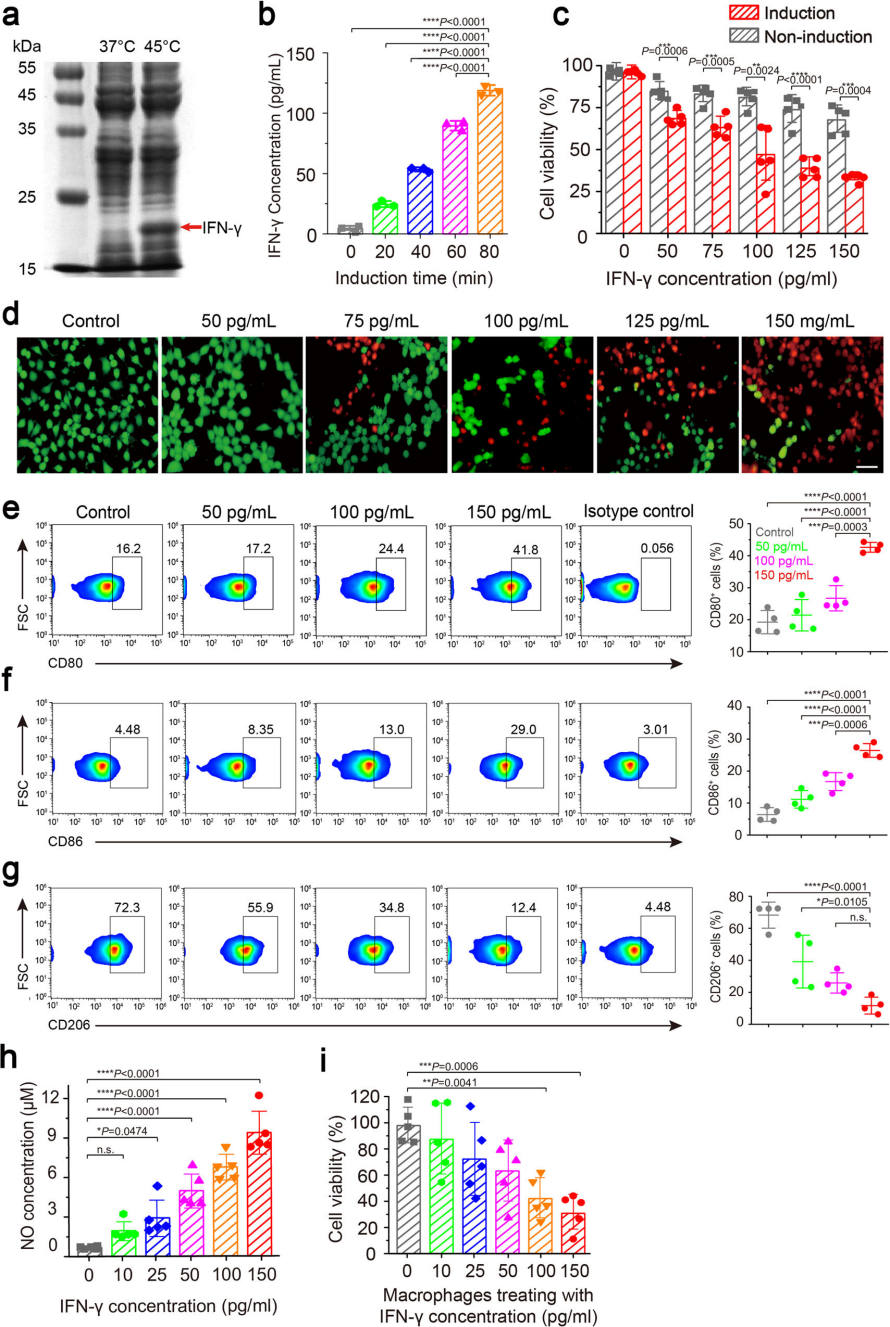
In vitro characterization of URB. **a** Representative SDS-PAGE image of IFN-γ protein expressed in URB under different temperature. Images were representative of three experiments. **b** Quantitative analysis of IFN-γ protein content in bacterial culture medium under ultrasound irradiation with different time. *n* = 3 biologically independent samples per group. **c** Viability assay of cells treated with bacterial culture medium with different IFN-γ concentrations. *n* = 5 biologically independent cells samples per group (Statistical analysis was calculated by using independent *T* test). **d** Fluorescence microscopy image of breast cancer cells stained with Calcein-AM and PI after different IFN-γ levels bacterial culture medium treatment (scale bar = 20 μm). Images were representative of three experiments. **e** Flow cytometry analysis of M1 phenotype macrophages (CD80^+^) after different IFN-γ levels bacterial culture medium treatment. *n* = 4 biologically independent cells samples per group. **f** Flow cytometry analysis of M1 phenotype macrophages (CD86^+^) after different IFN-γ levels bacterial culture medium treatment. **g** Flow cytometry analysis of M2 phenotype macrophages (CD206^+^) after different IFN-γ levels bacterial culture medium treatment. **h** Quantitative analysis of NO concentrations in the cell culture medium of macrophages RAW 264.7 treated with different bacterial IFN-γ concentrations. *n* = 5 biologically independent cells samples per group. **i** Cytotoxicity of macrophages stimulated with different IFN-γ concentrations to breast cancer cells. MOI ratio = 5:1 (tumor cells:macrophages). *n* = 5 biologically independent cells samples per group. Data were presented as mean ± S.D. Statistical analysis was calculated by using one-way analysis of variance with a Tukey’s test (****P < 0.0001; ***P < 0.001; **P < 0.01; *P < 0.05;). Source data are provided as a Source Data file.

Next, we engineered one therapeutic URB by displacing the fluorescent mCherry gene with murine IFN-γ gene with N-terminal OmpA signal sequence in the thermal logic circuits. Agarose gel electrophoresis confirmed the presence of IFN-γ gene fragment in the plasmid (Supplementary Fig. 4). SDS-PAGE gel electrophoresis analysis revealed that IFN-γ proteins appeared only in the lysate of bacteria incubated at 45 °C for 30 min, but not in the lysate of bacteria incubated at 37 °C, confirming the production of therapeutic IFN-γ after thermal treatment (Fig. 3a). The western blot assay further confirmed the IFN-γ expression in both bacteria lysate and culture medium after heat treatment (Supplementary Fig. 5). Prolonging the ultrasound irradiation time at 45 °C from 20 min to 80 min increased the expression level of IFN-γ proteins, showing a time-dependent correlation (Fig. 3b). These results showed the designed therapeutic logic circuit can be strictly controlled and tuned by heating or ultrasound stimulation and their exposure duration, which is important for many therapeutic factors to execute their anti-tumor effects. Cell killing assay by CCK-8 kits revealed that the cell viability was 33.13 ± 2.40%, 38.93 ± 5.69%, 47.02 ± 16.51%, 63.09 ± 6.02% or 68.50 ± 4.16% or 95.90 ± 1.49% for 150 pg/ml, 125 pg/ml, 100 pg/ml, 75 pg/ml, 50 pg/ml or 0 pg/ml IFN-γ proteins in the bacterial supernatant, respectively. It was significantly lower than these cells incubated with the equivalent supernatant from non-induced bacteria (Fig. 3c). To test whether the IFN-γ proteins could be secreted from their host bacteria to kill tumor cells, the Calcein AM/PI staining assay was used to 4T1 breast cancer cells which were incubated with bacterial supernatant containing different concentrations of IFN-γ proteins from 50–150 pg/ml. Almost all the cells showed green fluorescence in the blank group, showing no dead cells. With the increase of IFN-γ proteins, the proportion of living cells (green fluorescence) decreased gradually, while the proportion of dead cells (red fluorescence) increased gradually (Fig. 3d).

IFN-γ can exert its cytotoxicity and immunoregulatory effects by activating Janus kinase 1 (JAK1) and signal transducers and activators of transcription1 (STAT1) signaling pathways^30–32^. The effect of IFN-γ expressed from URB on macrophage immune activation was further examined in vitro. The RAW 264.7 macrophages were incubated with IFN-γ expressed by bacteria for 48 h, revealing a similar effect as the traditional IFN-γ in promoting the generation of proinflammatory M1 phenotype macrophages, with about 29.0% of macrophages staining positive for CD86 and 41.8% for CD80 at 150 pg/mL bacterial IFN-γ (Fig. 3e, f). Also, the significantly decreased expression of M2 phenotype marker CD206 could be observed on the macrophages (Fig. 3g), confirming that IFN-γ released from the URB can induce the macrophage polarization from M2 to M1 phenotype. Meanwhile, the bacterial IFN-γ also triggered to produce higher level of nitric oxide (NO) in the macrophages in comparison with the control group (Fig. 3h). Furthermore, we detected the tumor cell killing efficacy of these activated macrophages stimulated by different concentrations of bacterial IFN-γ, finding that higher concentrations of IFN-γ were used to stimulate macrophages, stronger cytotoxicity these activated macrophages would be (Fig. 3i). Together, the above results demonstrated that the IFN-γ produced from bacteria could effectively activate macrophages.

### Tumor targeting of ultrasound-responsive bacterium

**Fig. 4:**
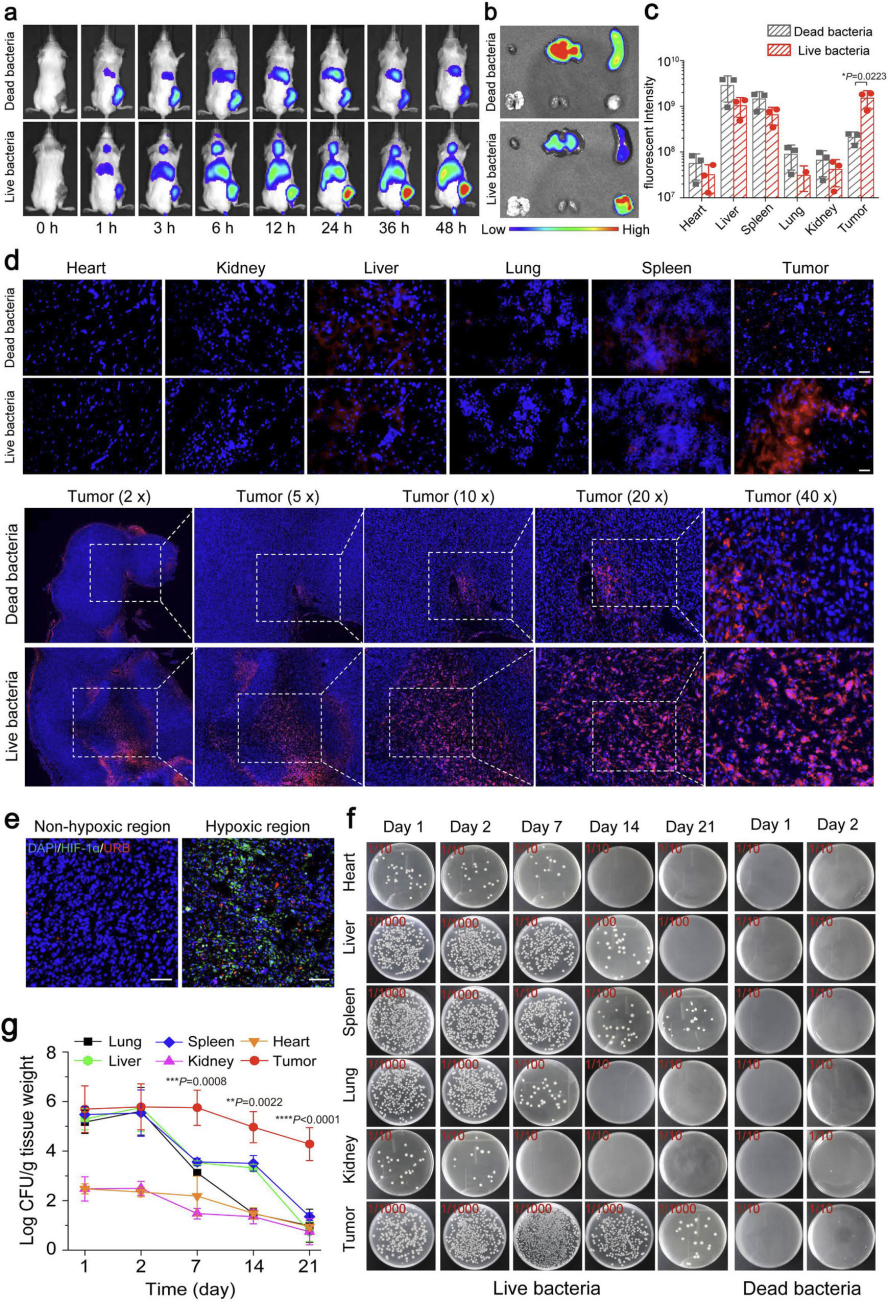
Tumor targeting of URB. **a** In vivo fluorescence imaging of tumor-bearing mice different time after intravenous injection of 1 × 10^7^ CFU of DiR-stained live URB or heating-inactivated URB. Images were representative of three experiments. **b**, **c** The intensities of fluorescence imaging (**b**) and corresponding fluorescence signal (**c**) of tumors and major organs of tumor-bearing mice 48 h after intravenous injection of 1 × 10^7^ CFU of DiR-stained live URB or heating-inactivated URB. *n* = 3 biologically independent animals per group. (Statistical analysis was calculated by using independent *T* test.) **d** Fluorescence microscopy images of tumors and major organs of tumor-bearing mice 48 h after intravenous injection of 1 × 10^7^ CFU of DiR-stained URB (scale bar = 25 μm). Images were representative of three experiments (*n* = 3 animals per group). **e** Immunofluorescence staining of tumor slices from peritumoral region (left) and central region (right). DAPI stands for the nuclei of tumor cells, red for DiR-stained live URB and green for HIF-1α highly expressed cells. Scale bar = 50 μm. Images were representative of three experiments (*n* = 3 animals per group). **f** Photographs of solid LB agar plates of bacterial colonization in tumors and major organs collected from tumor-bearing mice at different time points after intravenous administration of 1 × 10^7^ CFU of URB. **g** Quantification of bacterial colonization in major organs and tumors collected from tumor-bearing mice at different time points after administration of 1 × 10^7^ CFU of live URB (Asterisks: Tumor vs every other organs). *n* = 3 biologically independent animals per group at each time point. Data were presented as mean ± S.D. Statistical analysis was calculated by using one-way analysis of variance with a Tukey’s test (****P < 0.0001; ***P < 0.001; **P < 0.01; *P < 0.05;). Source data are provided as a Source Data file.

In order to detect the tumor-targeting property of these engineered bacteria, the DiR-labelled live URB or dead URB treated with 65 °C for 30 min were injected intravenously into the 4T1 tumor-bearing mice and imaged by an IVIS spectrum imaging system at different time. As shown in Fig. 4a, the live URB revealed the efficient tumor-homing ability in the 4T1 tumor, emitting strong fluorescence at 6 h after injection. Increasing fluorescence at the tumor site was observed along with time from the mice received live DiR-labelled URB administration, but not from the control mice injected with the dead URB. 48 h after administration, mice were euthanized to analyze the fluorescence signals in various organs (Fig. 4b). The results showed that the distribution of dead URB are mainly in liver and spleen. By contrast, the accumulation of the live URB was higher in the tumor site, and less in the liver than the control group. The quantitative analysis revealed a five-fold increase of relative fluorescence intensity in the tumor of live URB group compared with dead URB, which is consistent with the results of in vivo fluorescence imaging (Fig. 4c).

Subsequently, the targeting efficiency of DiR-labelled URB to the tumor was determined by fluorescence microscope. As showed in Fig. 4d, the red fluorescence signal, which represents the distribution of DiR-labelled live URB, mainly appeared in the tumor tissues but not in the heart, kidney and lung, and a bit in the liver and spleen. By contrast, only a little of red fluorescence could be observed in the tumor for DiR-labelled dead URB. Interestingly, the red fluorescence of DiR-labelled live URB were largely distributed in the center of tumor which represents the hypoxic and necrotic region due to the insufficient nutrient supply. Different from the live URB, the dead URB mainly appeared in the peritumoral zone. Immunofluorescence staining assay confirmed that URB localized in the region where the HIF-1α highly expressed, but not in the region where the tumor was not hypoxic (Fig. 4e). These results illustrated that the live URB rather than the dead URB could be efficiently accumulated in the tumor site and penetrated the tumor hypoxic and necrotic region. It may be the chemoreceptors on the surface of *E. coli* sense the nutrient-rich and low-oxygen tumor microenvironment, promoting them to migrate actively in the tumor region^33,34^. In order to further explore the behaviors of these engineered URB after intravenous injection, 4T1-bearing mice received URB at the dose of 1 × 10^7^ colony-forming units (CFU) per mouse via intravenous injection and then sacrificed at 1, 2, 7, 14, and 21 days after injection. Various organs and tumor were collected, homogenized, serially diluted (10–10000 fold), and incubated on Luria–Bertani (LB) plates containing 100 μg/ml ampicillin. The colony counts in each plate revealed that URB were gradually eliminated from heart, liver, spleen, lung and kidney. However, the colony counts of tumors showed exponential growth along with the time, achieving the peak value after seven days and then decreasing gradually (Fig. 4f, g). There was not bacterial colony formation from any tissue homogenates when the dead URB were used for intravenous injection into these mice. Collectively, these results provided evidence for their capability of the engineered bacteria to home, penetrate and colonize in the tumors, attributing to the hypoxic, immunosuppressive, and biochemically unique tumor microenvironment^35–37^.

### In vivo experiment of ultrasound-responsive bacterium-mediated tumor immunotherapy

**Fig. 5:**
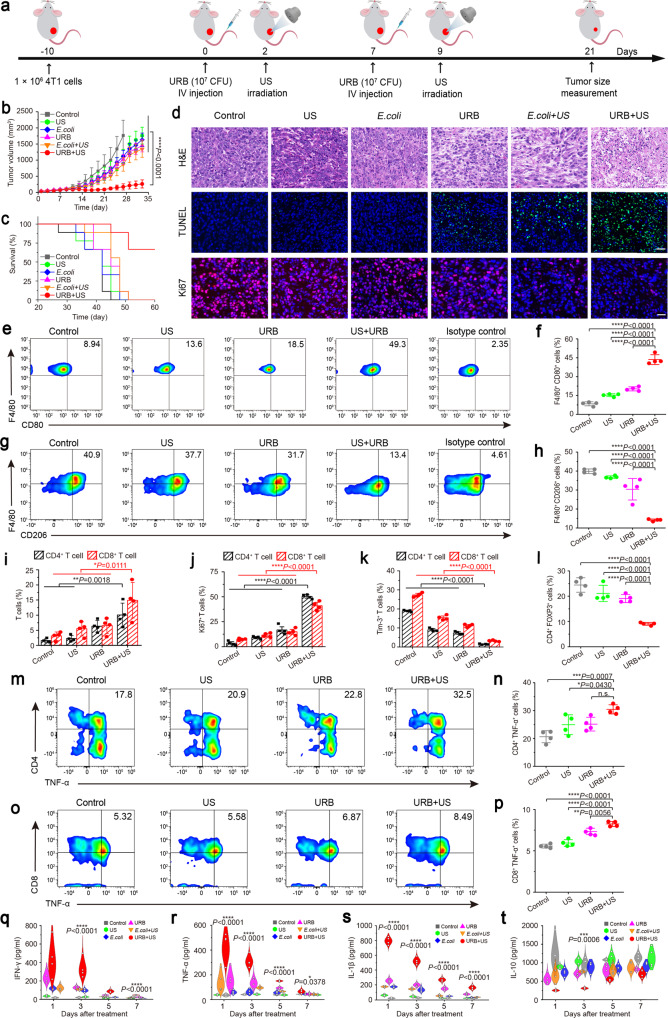
URB-mediated tumor immunotherapy. **a** Schematic illustration of URB-mediated tumor immunotherapy to inhibit tumor growth in a 4T1 subcutaneous tumor transplantation mouse model. **b** Tumor growth curve of tumor-bearing mice treated with different treatments. *n* = 9 biologically independent animals per group. (Asterisks: URB + US vs every other group). **c** Survival curves for different treatment groups. **d** H&E-stained, TUNEL-stained and Ki67-stained tumor slices for each group. Scale bar = 100 μm. Images were representative of three experiments (*n* = 3 animals per group). **e**, **f** Flow cytometric analysis and quantification of M1 macrophages (CD80^+^F4/80^+^) in tumor. **g**, **h** Flow cytometric analysis and quantification of M2 macrophages (CD206^+^F4/80^+^) in tumor. **i** Flow cytometric quantification of CD4^+^ T cell (CD4^+^CD3^+^) and CD8^+^ T cells (CD8^+^CD3^+^) in tumor. **j**, **k** Flow cytometric quantification of Ki67^+^ (**j**) and Tim-3^+^ (**k**) in CD4^+^ and CD8^+^ T cells in tumor. **l** Flow cytometric quantification of Treg (FOXP3^+^CD4^+^) cells in tumor. **m**, **n** Flow cytometric analysis and quantification of functional CD4^+^ T cells (TNF-α^+^CD4^+^) in tumor. **o**, **p** Flow cytometric analysis and quantification of functional CD8^+^ T cells (TNF-α^+^CD8^+^) in tumor. **q**, **t** IFN-γ (**q**), TNF-α (**r**), IL-1β (**s**) and IL-10 (**t**) levels in tumors at 1, 3, 5, 7 days after different treatments (Asterisks: URB + US vs every other group). For Fig. 5 (**e**–**t**), *n* = 4 biologically independent animals per group. Data were presented as mean ± S.D. Statistical analysis was calculated by using one-way analysis of variance with a Tukey’s test (****P < 0.0001; ***P < 0.001; **P < 0.01; *P < 0.05;). Source data are provided as a Source Data file.

To evaluate the anti-tumor effect of URB combined with focused ultrasound in vivo, unilateral 4T1 tumor-bearing mice model were treated following the therapeutic schedule in Fig. 5a. The mice were randomly separated into six groups and treated with saline (control), ultrasound alone (US), *E. coli* MG1655 harboring without the circuit (*E. coli*), URB harboring the circuit (URB), *E. coli* + ultrasound (*E. coli* + US) or URB + US group, respectively. 48 h after intravenous injection, ultrasound irradiation was performed to trigger the expression of IFN-γ. The treatment procedure was repeated once after seven days. As shown in Fig. 5b, c and Supplementary Figs. 6, 7, the volume of tumors in each group was similar on the day 1. However, URB + US group exhibited the strongest tumor inhibitory effect against tumor growth and the longest survival time. The average tumor volume of the mice treated with URB + US was less than 250 mm^3^, whereas that of other treated groups are more than 1000 mm^3^. Slight anti-tumor effect of US, *E. coli* or URB, or *E. coli* groups could be observed in our study, which may attribute to 30-min hyperthermia at 45 °C produced by US, the presence of lipopolysaccharide in the bacteria or nutrient competition due to bacterial growth^38–40^. The median survival time of mice in URB + US group increased to 60 days compared with 42 days for control group, which proved that application of URB combined with ultrasound irradiation could improve survival rate significantly. Compared with other groups, the treatment of URB + US resulted in apparent damages on the tumor cells (Fig. 5d, up row). The maximum degree of cell apoptosis was also found by terminal deoxynucleotidyl transferase-mediated dUTP nick end labeling (TUNEL) assay (Fig. 5d, middle row). Moreover, URB + US showed much less expression of Ki67 in immunofluorescence staining (Fig. 5d, low row), demonstrating significant reduced tumor proliferation potentials. The results of body weight demonstrated that systemic administration of URB did not cause severe side effects to mice (Supplementary Fig. 8).

As shown in Supplementary Fig. 10a, we can see that the tumor cells expressing MHC class I significantly increased in the URB + US group, achieving 39.35 ± 2.53% MHC I^+^ cell ratio in comparison with URB (29.5 ± 3.02%), US (28.75 ± 4.03%) and control (21.83 ± 9.13%) groups. Since IFN-γ can induce polarization of M1 macrophages^41^, we further explored whether the treatment activated the intratumoral immune response. Flow cytometry analysis of the polarization of tumor-associated macrophages (TAMs) demonstrated CD80^+^ macrophages greatly improved and CD206^+^ macrophages significantly decreased in the tumors treated with URB + US in comparison with control, only US and only URB groups, indicating that URB + US treatment could effectively promoted the polarization of TAMs from M2 phenotype towards M1 phenotype (Fig. 5e–h, Supplementary Figs. 9, 10b). In addition, we found URB + US treatment markedly increased CD4^+^ and CD8^+^ T cells (Fig. 5i). The proliferation, exhaustion and activation of T cells were further analyzed and revealed that Ki67^+^CD4^+^ and Ki67^+^CD8^+^ T cell significantly increased in the URB + US group in comparison with URB, US and control groups (Fig. 5j, Supplementary Fig. 10c, d). Moreover, less Tim3^+^ CD4^+^ and Tim3^+^CD8^+^ T cells which indicate of T cell exhaustion could be observed in the URB + US group than URB, US and control groups (Fig. 5k, Supplementary Fig. 10e, f). By contrast, FOXP3^+^CD4^+^ T cells of tumors treated with URB + US was much less than those of the control, US and URB groups (Fig. 5l, Supplementary Fig. 10g). As shown in Fig. 5m–p and Supplementary Fig. 11, comparing with the control groups, treatment with URB + US not only increased the number of functional CD4^+^ T cells in tumor, including IFN-γ^+^CD4^+^ (Supplementary Fig. 11a) and TNF-α^+^CD4^+^ (Fig. 5m, n) T cells, but also elevated the number of functional CD8^+^ T cells, such as IFN-γ^+^CD8^+^ (Supplementary Fig. 11b), TNF-α^+^CD8^+^ (Fig. 5o, p) and GranzymeB^+^CD8^+^ (Supplementary Fig. 11c) T cells. Moreover, significantly higher IFN-γ (Fig. 5q), TNF-α (Fig. 5r) and IL-1β (Fig. 5s) levels but less IL-10 (Fig. 5t) levels could be detected in the URB + US-treated tumors after 1, 3, 5 and 7 days. Thus, our data suggested that URB combined with ultrasound could effectively activate anti-tumor immunity through inducing the expression of IFN-γ. Some previous studies have used IFN-γ for tumor treatment and found that the infiltration of lymphocytes such as CD4^+^ T cells, CD8^+^ T cells, and macrophages in the tumor microenvironment increased significantly^42^. The significantly reduced ability of TAM to produce arginase-1 and iNOS was also demonstrated after IFN-γ treatment^43^. All these data suggested that IFN-γ could promote M1 macrophages in the tumor microenvironment, thereby improving the efficacy of tumor immunotherapy.

### In vivo experiment of ultrasound-responsive bacterium-mediated distant tumor and metastasis treatment

**Fig. 6:**
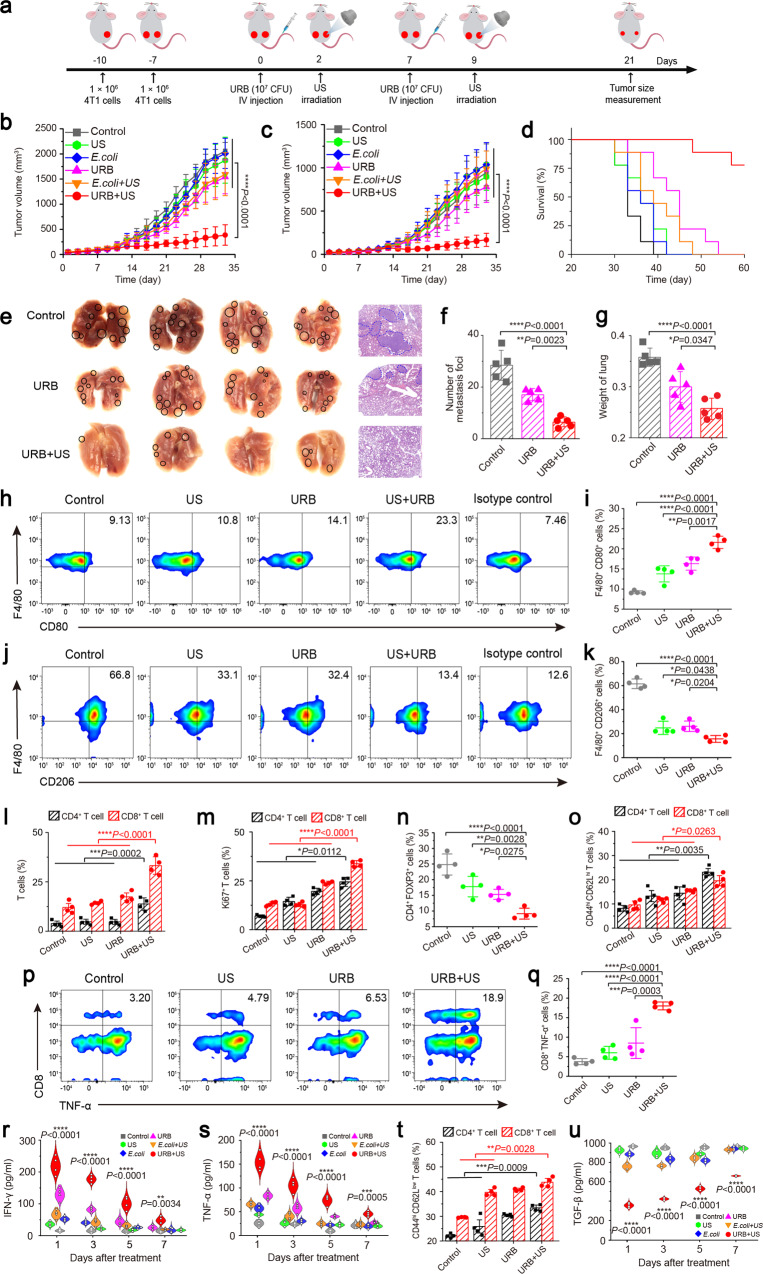
URB inhibited distant tumor growth and metastasis. **a** Schematic showing the treatment schedule in a distant tumor mouse model. **b** Primary tumor growth curve of mice treated with different treatments. *n* = 9 biologically independent animals per group (Asterisks: URB + US vs every other group). **c** Distant tumor growth curve of mice treated with different treatments (Asterisks: URB + US vs every other group). **d** Survival curves for different treatment groups. **e** Lung photographs and corresponding H&E-stained lung slices collected from the different group mice after treatment. **f** The number of metastasis foci in the lung of different groups (*n* = 5). **g** The weight of the lung of different groups (*n* = 5). **h**, **i** Flow cytometric analysis of M1 macrophages (CD80^+^F4/80^+^) in spleen. **j**, **k** Flow cytometric analysis of M2 macrophages (CD206^+^F4/80^+^) in spleen. **l** Flow cytometric quantification of CD4^+^ T cell (CD4^+^CD3^+^) and CD8^+^ T cell (CD8^+^CD3^+^) in spleen. **m** Flow cytometric quantification of Ki67^+^ in CD4^+^ and CD8^+^ T cells in spleen. **n** Flow cytometric quantification of Treg (FOXP3^+^CD4^+^) in spleen. **o** Flow cytometric quantification of central memory CD4^+^ and CD8^+^T cell in spleen. **p**, **q** Flow cytometric analysis and quantification of functional CD8^+^ T cells (TNF-α^+^CD8^+^) in spleen. **r** IFN-γ levels in serum at 1, 3, 5, 7 days after treatments. (Asterisks: URB + US group vs every other group). **s** TNF-α levels in serum at 1, 3, 5, 7 days after treatments (Asterisks: URB + US group vs every other group). **t** Flow cytometric quantification of effector memory CD4^+^ and CD8^+^T cell in distant tumor. **u** TGF-β levels in distant tumors at 1, 3, 5, 7 days after treatments (Asterisks: URB + US group vs every other group). For Fig. 6 (**h**–**u**), *n* = 4 biologically independent animals per group. Data were presented as mean ± S.D. Statistical analysis was calculated by using one-way analysis of variance with a Tukey’s test (****P < 0.0001; ***P < 0.001; **P < 0.01; *P < 0.05;). Source data are provided as a Source Data file.

Since the anti-tumor immunity of URB could be effectively activate by ultrasound in vitro, we next wondered whether the immune response triggered by URB combined with ultrasound can inhibit the growth of distant tumors. Bilateral 4T1 tumor-bearing mice model were established to explore the treatment-elicited abscopal (left) therapeutic effect by treating the primary (right) tumor. Firstly, we have detected the gene expression in the bilateral tumor-bearing mice model. As Supplementary Fig. 12a showed, the expression of fluorescent protein could be observed in the US-irradiated tumor (right) but not in the non-irradiated tumor (left). The fluorescent signals from US-irradiated tumor increased gradually along with the time (up to seven days). The ex vitro imaging of heart, lung, liver, spleen, kidney and tumor after 12 and 48 h further confirmed that US irradiation could induce the in-situ expression of mCherry gene (Supplementary Fig. 12b, c). As depicted in Fig. 6a, 4T1 cancer cells were inoculated at day −10 (right) and day −7 (left), respectively. A week later when the right flank tumors were about 100 mm^3^ (day 0), the engineered URB at 10^7^ CFU were systematacially administrated by tail vein and followed by ultrasound irradiation at two days later (day 2). As expected, significant anti-tumor effects were observed in the primary tumors received with ultrasound irradiation and URB administration (Fig. 6b, Supplementary Figs. 13, 14). Interestingly, the distant tumors from URB + US group were also greatly inhibited in comparison with control, US, *E. coli*, URB, *E. coli* + US groups (Fig. 6c, Supplementary Figs. 13, 14). The survival rate of the mice treated with URB + US was greatly improved, with more than 80% of them still alive on 60^th^ day post the tumor inoculation (Fig. 6d). Notably, only very few metastatic foci were observed in the lungs of mice treated with URB + US (Fig. 6e–g), suggesting effective inhibition of lung metastasis. In addition, we also demonstrated that systemic administration of URB did not cause severe side effects to mice by body weight monitoring (Supplementary Fig. 15). All of these data indicated URB + US resulted in a robust anti-tumor immune effect to protect mice from distant tumors and metastasis.

To understand the mechanism of the anti-tumor systemic effects triggered by URB plus ultrasound, immune cells in the spleen were assessed on the 7^th^ day after the first treatment by bilaterally 4T1 cancer model. The percentage of M1 phenotype macrophages (CD80^+^) and M2 phenotype macrophages (CD206^+^) in spleens was evaluated. The results showed that the proportion of CD80^+^ macrophages in the URB + US group was 21.6 ± 1.53 %, with a significantly increase compared with saline control group (9.22 ± 3.78%) (Fig. 6h, i, Supplementary Figs. 16, 17a). In contrast, the proportion of CD206^+^ macrophages in spleen were significantly decreased to 15.58 ± 2.83% (Fig. 6j, k). Specifically, the percentage of CD4^+^ and CD8^+^ T cells in the URB + US group occupied 13.75 ± 3.13% and 33.23 ± 4.44% with an increase by nearly 3.6 and 2.7 times in comparison with the control group, respectively (Fig. 6l). Further analysis found a significant increase of proliferative CD4^+^ (Ki67^+^CD4^+^) and CD8^+^ T cells (Ki67^+^CD8^+^), but a significant decrease of Treg (FOXP3^+^CD4^+^) in the spleen of mice treated with URB + US (Fig. 6m, n, Supplementary Fig. 17b–d). To further analyze the immunological memory effect, we detected the central memory T cell population in spleen and found that treatment with URB + US significantly increased the number of central memory CD8^+^ T cells (CD3^+^CD8^+^CD44^+^CD62L^+^) and central memory CD4^+^T cells (CD3^+^CD4^+^CD44^+^CD62L^+^) (Fig. 6o, Supplementary Fig. 18). The functional CD8^+^ T cells including GranzymeB^+^CD8^+^ cells (Supplementary Fig. 17e), IFN-γ^+^CD8^+^ cells (Supplementary Fig. 17f) and TNF-α^+^CD8^+^ cells (Fig. 6p, q) had the largest ratios in the spleens of mice treated with URB + US in comparison with other groups. These activated T cells led to a marked increase in IFN-γ (Fig. 6r) and TNF-α (Fig. 6s) level in blood.

Next, the proliferative and functional T cell population in the distant tumors were also detected to clarify the mechanism of growth inhibition of distant tumors. Data from flow cytometry analysis of these T cells by Ki67, TNF-α, IFN-γ and Gramzyme B markers confirmed significantly increased proliferative and functional CD4^+^ and CD8^+^ T cells in URB + US group (Supplementary Figs. 19a, b, 20a–e). Notably, Tim-3^+^CD4^+^ and Tim-3^+^CD8^+^ T cell population significantly decreased, showing there were less exhausted T cells (Supplementary Fig. 19c, d). Moreover, CD3^+^CD8^+^CD44^+^CD62L^-^ and CD3^+^CD4^+^CD44^+^CD62L^-^ cell populations in distant tumors significantly increased in the URB + US-treated distant tumors, indicating that there were more effector CD4^+^ and CD8^+^ memory T cells (Fig. 6t, Supplementary Fig. 21). To investigate the change of immunosuppressive microenvironment, ELISA was used to detect the levels of TGF-β in the distant tumors. Results showed that treatment with URB + US significantly decreased the TGF-β levels in distant tumors in comparison with other groups (Fig. 6u). Collectively, these results indicated that the URB + US treatment could trigger the systemic anti-tumor immunity to inhibit the distant tumor growth and metastasis^44–46^.

**Fig. 7:**
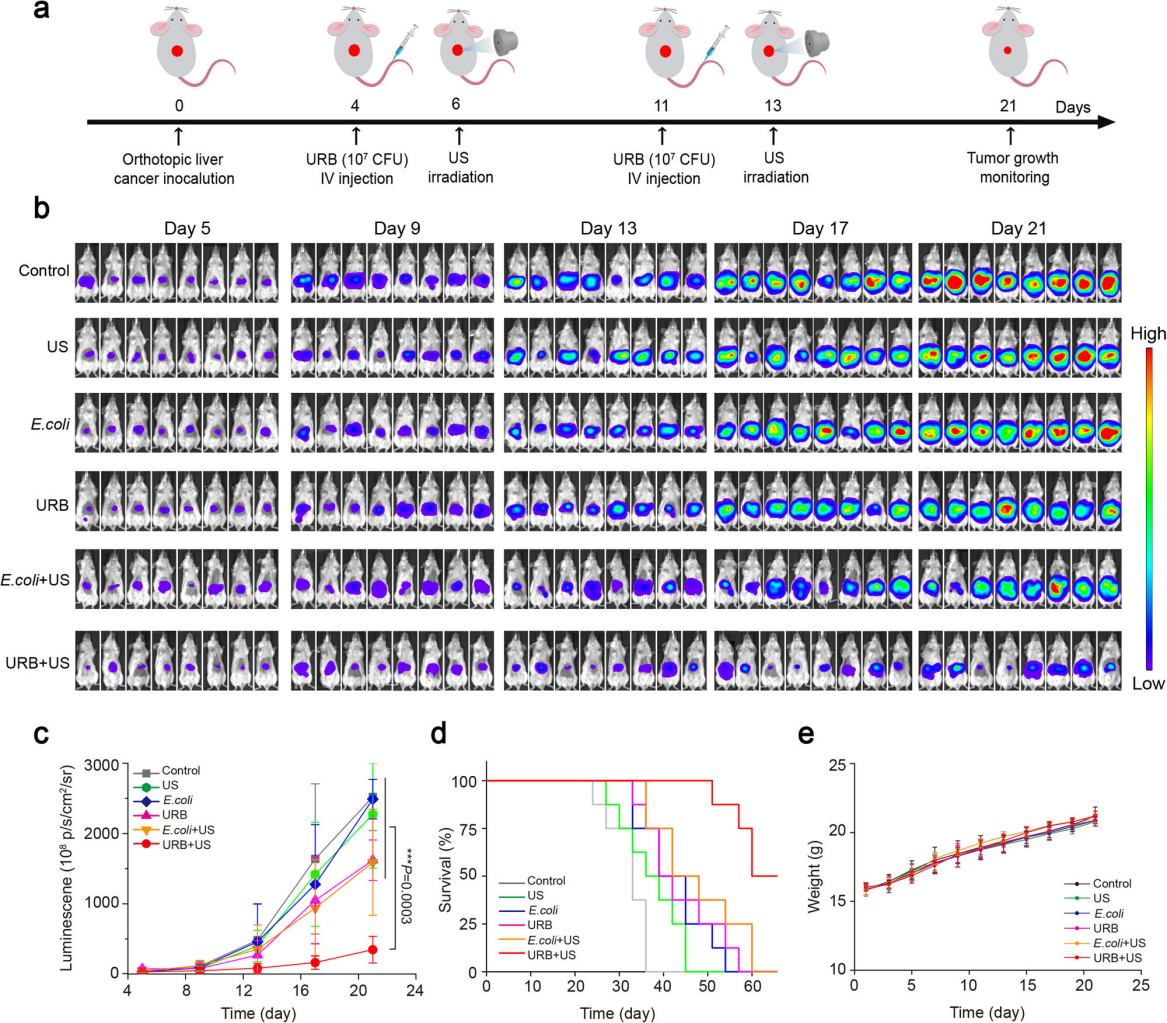
Growth inhibition effect of orthotopically transplanted liver tumor. **a** Schematic showing the treatment schedule in the orthotopically transplanted liver tumor model (*n* = 8). **b** Representative bioluminescence images of liver tumor-bearing mice in different groups. **c** Bioluminescence quantification of liver tumor-bearing mice treated with different treatments. *n* = 8 biologically independent animals per group (Asterisks: URB + US group vs every other group). **d** Survival curves of tumor-bearing mice for different treatment groups. **e** Weight change of mice treated with different treatments. Data were presented as mean ± S.D. Statistical analysis was calculated by using one-way analysis of variance with a Tukey’s test (****P < 0.0001; ***P < 0.001; **P < 0.01; *P < 0.05). Source data are provided as a Source Data file.

To explore the anti-tumor efficacy of URB + US in the deep-seated tumor, we established a mouse orthotopically transplanted liver tumor model by intrahepatic injection of H22-Luc cells. As shown in Fig. 7a, four days after H22-Luc cells were inoculated in the liver of mice, the *E. coli* or engineered URB were administrated intravenously into the mice. Still two days later, ultrasound irradiation was applied to irradiate the tumors. From the data shown in Fig. 7b, we can see that similar fluorescent signals could be observed in each group on the day 5, showing the successful development of orthotopically transplanted H22-Luc liver tumors. No significant tumor inhibitory effects were found in the control, only US and *E.coli* groups. Slight tumor inhibitory effects were observed in the URB and *E.coli* + US groups. However, URB + US group exhibited strongest tumor inhibitory effect (Fig. 7b, c). The median survival time of mice in URB + US group increased to 63 days compared with 33 days for control group (Fig. 7d). These data indicated URB + US could significantly inhibit the orthotopic liver tumor growth and improve the survival rate of tumor-bearing mice. Meanwhile, no significant change of the weight of the mice was found in the URB + US-treated mice (Fig. 7e).

In order to further explore the biosafety of this therapeutic strategy, the changes of mouse body weight and histopathologic features of major organs were evaluated. No significant body weight changes of mice were found in the different treatment (Supplementary Fig. 5), demonstrating the good biosafety of the URB-mediated immunotherapy. In our study, cell damage was not observed in the H&E staining slices of various organs of different group mice (Supplementary Fig. 22). In spite of this, it is necessary to optimize the ultrasonic parameters to keep the constant temperature at 45 °C for in vivo application since too high temperature (>50 °C) would strongly associated with tissue damage over 30 s. The blood biochemistry and blood cell counts were recorded, showing that all of indexes of the URB + US group fell within the normal ranges. Only creatinine and direct bilirubin increased and platelet (PLT) decrease in the URB + US group when comparing with saline control (Supplementary Tables 1, 2). Because URB had less accumulation in normal tissues such as organs and blood. Previous studies used a similar bacteria strain to our study, *E. coli* MG1655, and confirmed that the strain was safe and did not affect the homeostasis of the body^21^.

## Discussion

In this study, we have engineered an ultrasound-responsive bacterium (URB) for remote control of the expression of cytokine IFN-γ gene by focused ultrasound-induced hyperthermia. The genetically engineered bacterium (*E. coli* MG1655) could selectively accumulate and grow at the hypoxic region of tumors upon systemic administration, making it possible to locally produce cytokine IFN-γ under acoustic irradiation. As demonstrated in Figs. 5b, c and 7b–d, URB exhibited significant tumor-suppressive effects upon their exposure to ultrasound in both the subcutaneously transplanted breast tumor model and the orthotopically transplanted liver tumor model, greatly prolonging the survival time of the tumor-bearing mice. It is worth mentioning that treatment with URB + US also exhibited the strong tumor inhibitory effect against distant tumor growth in the bilateral 4T1 tumor-bearing mice model and reduced the number of lung metastatic colonies (Fig. 6c, e–g). It may mainly contribute to the anti-tumor immune response activated by IFN-γ. As showed in Fig. 5e–i, markedly increased CD4^+^ T cells, CD8^+^ T cells and CD80^+^ macrophages while significantly decreased CD206^+^ macrophages could be observed in the tumors treated with URB + US, indicating that URB + US treatment could effectively repolarize TAMs towards M1 phenotype and thus improving the macrophages phagocytosis and T cell activation.

It needs to be pointed out that the in vivo toxicity is still a concern for the clinical translation of bacteria-mediated immunotherapy. Our biosafety study showed URB + US treatment did not produce significant tissue damage and body weight changes to these mice (Supplementary Fig. 8 and Supplementary Fig. 22). The good biosafety of URB + US treatment not only may contribute to the targeted expression of cytokines which reduces the risk of cytokine storms, but also to the rapid removal of bacteria by normal organs. Scalability is also a critical issue that needs to be addressed for clinical application. Previous study reported a thermosensitive therapeutic bacterium and triggered the in-situ TNF-α expression of bacteria by near-infrared light^21^. However, it is difficult to act on deep-seated tumors in live animals due to the poor tissue penetration of near-infrared light. By contrast, ultrasound has higher tissue permeability and can irradiate deeper tumors seated in liver, pancreas, prostate or ovary. Our results also confirmed that URB + US treatment could be applied to the orthotopically transplanted liver tumor (Fig. 7). Although significantly enhanced anti-tumor efficacy and good safety of URB + US treatment have been seen in this study, some steps are still necessary to be identified for the future clinic use. For example, the safer non-pathogenic bacteria or attenuated bacteria in which virulence genes were deleted should be used. As an alternative, it is possible to achieve the acoustic control of therapeutic gene expression for anti-tumor therapy by using of T cells or NK cells. In fact, literatures have reported that T cells could be engineered with thermal sensitive gene circuits to realize intratumoral cytokine or protein expression for anti-tumor therapy^47–49^. In addition, the expression level of therapeutic genes needs to be further optimized for achieving optimal therapeutic outcomes in the future clinical application.

In summary, we have developed an ultrasound-responsive bacterium (URB) which can express therapeutic genes in an acoustic control of gene expression manner. Our study reveals the URB carrying cytokine IFN-γ gene had good tumor-targeting capability and reliable tumor-suppressive effect upon their exposure to ultrasound for expressing therapeutic genes. Significant anti-tumor growth and metastasis were demonstrated. In conclusion, we developed a high spatiotemporally controllable gene expression strategy in deep-seated tumors through the combination of URB and focused ultrasound.

## Methods

This research complies with all relevant ethical regulations approved by Shenzhen Institute of Advanced Technology, Chinese Academy of Sciences.

### Materials

The IFN-γ gene and OmpA peptide gene sequence was obtained from GenBank and synthesized by Sangon bioengineering Co.(Shanghai, China). The pBV220 plasmid was purchased from Miaoling Bioscience & Technology Co.(Wuhan, China). *E. coli* DH5α strain and *E. coli* MG1655 strain were purchased from Angyubio Biotechnology Co. (Shanghai, China). The mouse breast cancer cells (4T1) were obtained from American Type Culture Collection. Dulbecco’s modified Eagle’s medium (DMEM) culture medium, fetal bovine serum (FBS), penicillin and streptomycin solution, trypsin containing 0.25% EDTA were purchased from Gibco (USA). 1,1-dioctadecyl-3,3,3,3-tetramethylindotricarbocyanine iodide (DiR), live & dead cell viability detection kit and CCK-8 assay kit were obtained from Beyotime Biotechnology Co. (Shanghai, China). Murine IFN-γ, TNF-α, IL-10, IL-1β and TGF-β enzyme-linked immunosorbent assay (ELISA) kits were obtained from Dakewe Bioengineering Co. (Shenzhen, China).

### Preparation of ultrasound-responsive bacterium

The IFN-γ gene with N-terminal OmpA secretion signal sequence was inserted into a pBV220 plasmid through EcoRI and SalI enzyme digestion and ligation reactions to generate the IFN-γ-expressed thermosensitive plasmid (pBV220-IFN-γ). The recombinant plasmid was transformed into *E. coli* DH5α competent cells by chemical transformation protocol. The construction of pBV220-IFN-γ plasmid was confirmed by enzyme digestion. Also, the mCherry-expressed thermosensitive control plasmid (pBV220-mCherry) was constructed to facilitate the detection of gene expression. The pBV220-IFN-γ or pBV220-mCherry plasmids were transformed chemically to the *E. coli* MG1655 strain to obtain ultrasound-responsive bacterium (URB). The resulting URB solution was plated on LB solid plate with ampicillin and incubated at 37 °C for 12 h. URB colonies were picked out and amplified in LB medium at 30 °C, 220 rpm overnight. Afterward, the URB solution was diluted by 100-folds into LB medium and further grown to the OD_600_ = 0.4–0.6 for further experiments.

### Thermal-induced and ultrasound-induced mCherry expression

The URB transformed with pBV220-mCherry plasmid were incubated at 37 °C or 45 °C for a given time and further incubation for 6 h at 37 °C. The IVIS Spectrum was used to detect the fluorescence signal of bacteria. To test the feasibility of ultrasound-induced mCherry gene expression, the URB was added to a 96-well plate (200 μl each well). Ultrasound with acoustic pressure of 2.82 MPa, 3.17 MPa, 3.52 MPa, 3.88 MPa, 4.22 MPa and 4.93 MPa were used to generate heating to induce gene expression. The ON/OFF ultrasound irradiation procedure was adjusted to keep the temperature constant. Other ultrasound parameters were as follows: frequency = 960 Hz, pulse period = 150 ms, pulse width = 100 ms, irradiation time = 30 min. The temperature of URB solution was monitored by an infrared thermal imager. In the following in vitro experiments, we will use this parameter (4.93 MPa acoustic negative pressure, 3 s ON and 5 s OFF irradiation time) which could keep the irradiated bacterium solution at 45 °C constant temperature. After ultrasound irradiation, the fluorescence signal of URB solution was observed at different time points (from 1 day to 7 day) after irradiation. To examine the penetration of the ultrasonic stimulation, 1% agar phantom was made and inserted a tube filled with URB, keeping the tube 10 cm away from the transducer. Then the tube was irradiated by ultrasound for 30 min. The fluorescence signal of URB was detected by IVIS Spectrum. To detect the activity of bacteria after ultrasound irradiation, URB treated with ultrasound or heating (45 °C) was plated on the LB plate to record colonies or added to 96 well to observed OD_600_ change during 48 h culture.

Due to the difference in thermal properties and ultrasonic energy absorption of experimental samples (bacterial solution and mouse tissue), it is still necessary to explore the specific ultrasonic parameters to raise the temperature of animal tissues. Balb/c mice (female, 6–8 weeks) were anesthetized with isoflurane and focus ultrasound was used to irradiate mice right thigh. Ultrasound with acoustic negative pressure of 2.82, 3.17, 3.52, 3.88, 4.22 and 4.93 MPa were used for irradiation, respectively. The ON/OFF ultrasound irradiation procedure was adjusted to keep the temperature constant. The temperature of mice thigh was monitored by an infrared thermal imager. In the following in vivo experiments, we will use this parameter (3.52 MPa acoustic negative pressure, 3 s ON and 5 s OFF irradiation time) which could keep the irradiated mice tissue at 45 °C constant temperature. In order to confirm the in vivo gene expression of URB, 1 × 10^7^ CFU of URB were injected into the Balb/c mice via the tail vein and ultrasound was used to irradiate the liver of mice at 3.52 MPa acoustic pressure for 30 min. Fluorescence signal of mice and the major organs were detected by IVIS at 6 h after ultrasound irradiation. As a control, the mice in the heat group were placed in an incubator at 45 °C for 30 min.

### Thermal-induced and ultrasound-induced IFN-γ expression

The URB transformed with pBV220-IFN-γ plasmid was cultured to OD_600_ 0.4–0.6 in 30 °C moved to 37 °C or 45 °C for 30 min. After that, the bacteria proteins were analyzed by SDS-PAGE to detect the IFN-γ expression in URB. Meanwhile, western blot was used to confirm the IFN-γ expression in bacteria lysate and culture medium. To determine the expression level of IFN-γ induced by ultrasound, URB was added to a 96-well plate and exposed to ultrasound (acoustic pressure = 4.93 MPa) for 20 min to 80 min. and the murine IFN-γ ELISA kit was used to quantitatively analyze the concentration of IFN-γ in bacterial culture medium.

### Cell cytotoxicity of IFN-γ expressed by ultrasound-responsive bacterium

Breast cancer 4T1 cells were seeded into a 96-well plate (1 × 10^4^ cells per well), and then incubated in 100 μl DMEM medium containing 10% FBS for 12 h. URB were used to induce and secrete the IFN-γ protein by ultrasound irradiation. The concentration of IFN-γ in ultrasound-induced URB culture supernatant was determined by ELASA kits and diluted to the indicated concentrations (from 0 to 150 pg/ml) with DMEM medium. The bacterial culture supernatant with corresponding concentrations of IFN-γ (0–150 pg/ml) was added into the tumor cells. The same volume of bacterial culture supernatant of URB which was not induced by ultrasound was used as the control. After incubating for 6 h, the tumor cells were washed with PBS for three times. CCK-8 kits were used to detect the cell cytotoxicity of IFN-γ expressed by URB according to the production illustration. Similarly, Calcein AM/PI staining was used to detect the 4T1 breast cancer cell viability after IFN-γ treatment. The fluorescence microscope was used to observe the fluorescent signal of tumor cells.

### Immunological activity of IFN-γ expressed by ultrasound-responsive bacterium

The immunological activity of IFN-γ expressed by URB was evaluated by macrophages activation. RAW 264.7 macrophages were seeded into a 96-well plate (8 × 10^3^ cells per well), and then incubated in 100 μl DMEM medium containing 10% FBS for 12 h. The bacterial culture supernatant with corresponding concentrations of IFN-γ (50–150 pg/ml) was added into the macrophages and incubated for 48 h. To detect the phenotype of macrophages, the treated RAW 264.7 macrophages were stained with corresponding antibodies: CD86-PE (Biolegend, Cat No. 105007, dilution ratio 1:100), CD80-BV421 (Biolegend, Cat No. 104725, dilution ratio 1:100) and CD206-APC (Biolegend, Cat No. 141707, dilution ratio 1:100) according to the manufacturer’s instructions and analyzed by flow cytometry. PE-Rat IgG2a (Biolegend, Cat No. 400507, dilution ratio 1:100), BV421-IgG (Biolegend, Cat No. 400935, dilution ratio 1:100) and APC-Rat IgG2a (Biolegend, Cat No. 400511, dilution ratio 1:100) were used for isotype control. After that, The NO detection kit (Beyotime, Cat No. S0021S) was used to detect the NO level in macrophage culture medium. Moreover, RAW 264.7 macrophages treated with different levels of IFN-γ were collected and used to co-incubate with tumor cells at a MOI ratio of 5:1 in 96-well plate for 12 h, and the tumor killing activity was evaluated with CCK-8 kit.

### Breast cancer model establishment

Balb/c mice were purchased from Vital River Laboratory Animal Technology Co. (Beijing, China). Animal experiments were approved by the Institutional Animal Care and Use Committee (IACUC) of the Animal Experiment Center of Shenzhen Institutes of Advanced Technology, Chinese Academy of Sciences(SIAT-IACUC-210831-HCS-YF-A2043). The tumor-bearing animals requires to be euthanized when the tumor burden reaches 2000 cm^3^ in volume. In some cases, this limit has been exceeded the last day of measurement and the mice were immediately euthanized. The tumor volume = tumor length × tumor width^2^ × 0.5. Mice were housed in a Specific-pathogen-free (SPF) facility at 23–25 °C on a 12-h light/dark cycle with enough food and water. The female Balb/c mice (4–6 weeks) were used to establish xenograft tumor model. 1 × 10^6^ 4T1 breast cancer cells in 100 μl DMEM medium were injected subcutaneously into the right thigh of the mice. The tumor volume was measured every two days after tumor implantation, and the treatment procedure was performed afterthe mice tumor volume reached to 100 mm^3^. The mice were anesthetized with 1–2% isoflurane in oxygen via a nose cone in experiments. All mice were euthanized by using carbon dioxide asphyxiation after experiments.

### Tumor targeting of ultrasound-responsive bacterium

The concentration of URB was adjusted to 1 × 10^8^ CFU/ml. DiR dye was added to the URB solution (30 μl/mL) and incubated at 37 °C for 45 min. The bacteria solution was washed with PBS until the supernatant was clear. The bacteria were injected into the tumor-bearing mice via the tail vein (1 × 10^7^ CFU per mouse) (*n* = 4). As the control, the dead URB were treated at 65 °C for 30 min. At the given time points, the mice were imaged by IVIS system (PekinEmer) to evaluate the biodistribution and tumor accumulation of bacteria. 48 h after the intravenous injection, tumor-bearing mice were sacrificed and the organs including heart, lung, liver, kidney, spleen and tumor were collected for the ex vivo fluorescence imaging. Meanwhile, the organs and tumor were cut into slices with 5 μm thickness, followed by staining with DAPI and FITC-labeled anti-HIF-1α antibody. To evaluate the number of URB in each organ (CFU per gram of tissue), different time after administration with URB at 1 day, 2 day, 7 day, 14 day and 21 day, organs of mice including heart, liver, spleen, lung, kidney and tumor were collected, weighed and homogenized in sterile PBS in ice. The tissue grinding solution was diluted from 10 to 10000 times with PBS. 100 μl tissue grinding solution was plated on the LB plate with 100 μg/ml ampicillin. The colonies formed after incubation for 12 h at 37 °C was counted.

### In vivo tumor treatment

The 4T1 tumor-bearing mice with approximate tumor volume of 100 mm^3^ were divided into six groups randomly (*n* = 9), including control group, US group, *E. coli* group, URB group, *E. coli* + US group and URB + US group. 1 × 10^7^ URB in 200 μl saline was injected intravenously into tumor-bearing mice in URB group and URB + US group. 1 × 10^7^ *E. coli* MG1655 was injected intravenously into tumor-bearing mice in *E. coli* group and *E. coli* + US group. The control group was injected with the 200 μl of saline. After 48 h of bacteria injection, the mice in US group, *E. coli* + US group and URB + US group were irradiated with the ultrasound for 30 min. The ultrasound parameter was as follows: acoustic pressure = 3.52 MPa, frequency = 960 Hz, pulse period = 150 ms, pulse width = 100 ms, ON 10 s/OFF 5 s. The treatment procedure was performed day 0 and day 7. Then tumor volumes and mice weights were recorded every 2 days. The calculation formula of tumor volume was: volume = (tumor length × tumor width^2^)/2. When the mice died, the survival time was recorded. After the mice died, the tumor were collected for H&E staining, TUNEL assay and Ki67 staining.

For flow cytometry analysis of intratumoral immune cells, the tumors were collected at seven days after treatment and cut into small pieces, then the tissue was digested with collagenase V (2 mg/ml, YEASEN) in HBSS solution for 4 h at 37 °C. Cells were then filtered through 70 μm cell strainer to obtain single-cell suspension and washed with FACS buffer. The single cell suspension was pre-blocked with CD16/32 antibodies (Biolegend, Cat No. 101319) for 30 min at 4 °C. Then the cells were stained with the following surface antibodies: CD45 (Biolegend, Cat No. 103127, dilution ratio 1:100), CD3 (Biolegend, Cat No. 100203, dilution ratio 1:100), CD4 (Biolegend, Cat No. 116019, dilution ratio 1:100), CD8 (Biolegend, Cat No. 100721, dilution ratio 1:100), Tim-3 (Biolegend, Cat No. 119703, dilution ratio 1:50), CD11b (Biolegend, Cat No. 101205, dilution ratio 1:100), F4/80 (Biolegend, Cat No. 123151, dilution ratio 1:100), CD80 (Biolegend, Cat No. 104725, dilution ratio 1:100), MHC II (Biolegend, Cat No. 107641, dilution ratio 1:100), CD206 (Biolegend, Cat No. 141707, dilution ratio 1:100), MHC I (Biolegend, Cat No. 114613, dilution ratio 1:100), CD44 (Biolegend, Cat No. 141707), CD62L424401103023, dilution ratio 1:50), CD62L (Biolegend, Cat No. 104435, dilution ratio 1:50) according to the manufacturer’s instructions. For the intracellular and intranuclear staining, after surface staining, the cell suspension was fixed and permeabilized with True-Nuclear™ Transcription Factor Buffer Set (Biolegend, Cat No. 424401) in accordance with the manufacturer’s protocol. The cells were stained with the following intracellular or intranuclear antibodies: IFN-γ (Biolegend, Cat No. 505831, dilution ratio 1:50), TNF-α (Biolegend, Cat No. 506307, dilution ratio 1:50), Granzyme B (Biolegend, Cat No. 372207, dilution ratio 1:50), Ki67 (Biolegend, Cat No. 652403, dilution ratio 1:100), FOXP3 (Biolegend, Cat No. 126419, dilution ratio 1:50) and analyzed by flow cytometry. The data were analyzed using FlowJo. The intratumor levels of IFN-γ, TNF-α, IL-1β and IL-10 were detected with ELISA kits (Dakewe) according to the instructions on the day 1, day 3, day 5 and day 7 after the treatment.

### Distant tumor and metastasis treatment

For the 4T1 distant tumor model establishment, 1 × 10^6^ 4T1 breast cancer cells were injected subcutaneously into the right thigh of Balb/c mice. On the third day after the inoculation, another 1 × 10^6^ 4T1 cells were injected into the left thigh of mice. After 10 day of the first inoculation, the mice were divided into six groups and treated as described above (*n* = 9). After 48 h of bacteria injection, the primary tumor of mice in US group, *E.coli* + US group and URB + US group were irradiated with the ultrasound for 30 min. The treatment procedure was performed day 0 and day 7. Tumor volume, body weight and survival time of mice were recorded continuously. For lung metastasis treatment, the treated mice (*n* = 5) were euthanatized on the 21st day by carbon dioxide asphyxiation and collected their lungs. The number of metastatic nodules in the lungs and the lung weight was recorded. The lung slices were then stained with H&E to observe the metastasis foci.

For flow cytometry analysis of immune cells in spleen and distant tumor, the spleens and distant tumors were collected at seven days after treatments and cut into small pieces, then the tissues were digested with collagenase V (2 mg/ml, YEASEN) in HBSS solution for 4 h at 37 °C. Cells were filtered through 70 μm cell strainer to obtain single cell suspension, and then analyzed by flow cytometer as stated above.

### Biosafety assay

Blood routine indexes, blood biochemical indexes and H&E staining of organs were used to evaluate the biosafety of URB. The tumor-bearing mice were randomly divided into six groups (*n* = 3) including control group, US group, *E.coli* group, URB group, *E.coli* + US group and URB + US group. The treated mice were sacrificed seven days after ultrasound irradiation. Blood samples were collected by eyeball extirpating. The organs were collected to perform H&E staining.

### Statistical analysis

All data were presented as mean ± SD. Independent T test was used for two groups compared. One-way ANOVA using the Tukey post-test for more than two groups compared. All statistical analyses were performed by SPSS 26.0. The results were significant when ***P < 0.001; **P < 0.01; *P < 0.05.

### Supplementary information

Supplementary Information

### Source data

Source Data

## Data Availability

The data that support the findings of this study are available within the Article, Supplementary Information, or Source Data file. Source data are provided with this paper.
