# Author Correction: Spatiotemporal control of engineered bacteria to express interferon-γ by focused ultrasound for tumor immunotherapy

**DOI:** 10.1038/s41467-024-48061-2

**Published:** 2024-04-30

**Authors:** Yuhao Chen, Meng Du, Zhen Yuan, Zhiyi Chen, Fei Yan

**Affiliations:** 1 The First Affiliated Hospital, Medical Imaging Centre, Hengyang Medical School, University of South China, Hengyang, Hunan 421001 China; 2 Institute of Medical Imaging, Hengyang Medical School, University of South China, Hengyang, Hunan 421001 China; 3 Faculty of Health Sciences, Centre for Cognitive and Brain Sciences, University of Macau, Taipa, Macau SAR, China; 4 Center for Cell and Gene Circuit Design, CAS Key Laboratory of Quantitative Engineering Biology, Shenzhen Institute of Synthetic Biology, Shenzhen Institute of Advanced Technology, Chinese Academy of Sciences, Shenzhen, 518055 China

**Keywords:** Protein delivery, Immunization, Cancer therapy

Correction to: *Nature Communications* 10.1038/s41467-022-31932-x, published online 2 August 2022

The original version of the manuscript contained errors in Figure 4 and in the Supplementary Information file. Original Figure 4 has now been replaced with the corrected version in the Article file (PDF and HTML versions) and the revised Supplementary Information file has been made available. In detail:

- In Figure 4f, the representative image of Kidney (Dead bacteria, Day 2) was incorrect and has been replaced.
- In Figure 4f, dilution ratios specified for the representative images of Liver (Live bacteria, Day 2 and Day 7), Spleen (Live bacteria, Day 7) and Tumor (Live bacteria, Day 2 and Day 7) were incorrectly labelled. The original values have been replaced with the correct ones.
- In Figure 4g, the graph for Kidney was incorrect and has been replaced with the correct one (as per values available in the Source Data file).
- In the Supplementary Information file, affiliation ‘1’ was incorrectly given. The correct affiliation has now been provided.
- In Supplementary Fig. 22, the representative images of Heart in the US group and *E. coli* group, Kidney in the *E. coli* group and URB group, and Lung in the *E. coli* group were incorrect and have now been replaced.

### Supplementary information

Updated Supplementary Information

