# Supplementary material for: Spatiotemporal control of engineered bacteria to express interferon-γ by focused ultrasound for tumor immunotherapy
Source: Nat Commun. 2022 Aug 2;13:4468. doi: 10.1038/s41467-022-31932-x (PMC9345953; doi:10.1038/s41467-022-31932-x)
Supplement: Supplementary file 1 — Supplementary Information [file 41467_2022_31932_MOESM1_ESM.pdf]

Supplementary Information

Spatiotemporal control of engineered bacteria to express interferon-γ by focused ultrasound for tumor immunotherapy

Yuhao Chen[1,2,3#], Meng Du[1,2#], Zhen Yuan [3],Zhiyi Chen[1,2*], Fei Yan [4*]

[1] The First Affiliated Hospital, Medical Imaging Centre, Hengyang Medical School, University of South China, Hengyang, Hunan, 421001, China

[2] Institute of Medical Imaging, Hengyang Medical School, University of South China, Hengyang, Hunan, 421001, China.

[3] Faculty of Health Sciences, University of Macau, Taipa, Macau SAR, China.

[4] CAS Key Laboratory of Quantitative Engineering Biology, Shenzhen Institute of Synthetic Biology, Shenzhen Institute of Advanced Technology, Chinese Academy of Sciences, Shenzhen, 518055, China.

*Corresponding Authors: Fei Yan, PhD, E-mail: fei.yan@siat.ac.cn; Zhiyi Chen, PhD, E-mail: zhiyi_chen@gzhmu.edu.cn

Supplementary figures

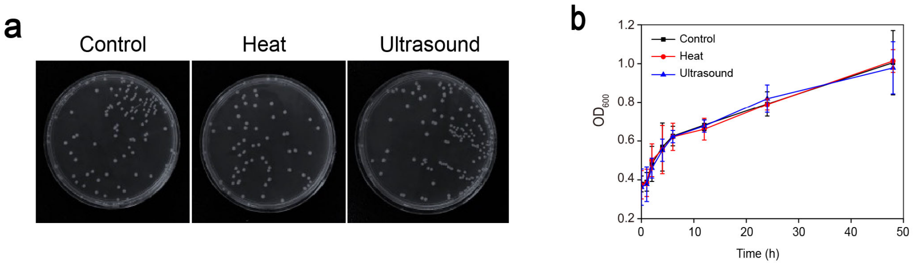

Supplementary Figure 1. Detection of bacteria activity. a: Representative photographs of solid Luria-Bertani (LB) agar plates of bacterial colonization after heating or ultrasound irradiation at 45°C for 30 min. b: Growth curves of URB treated with 45°C heating or ultrasound irradiation for 30 min. The untreated URB was used as the control. The number of bacteria was determined at OD_{600}. n = 3 biologically independent samples per group. Data were presented as mean ± S.D. Source data are provided as a Source Data file.

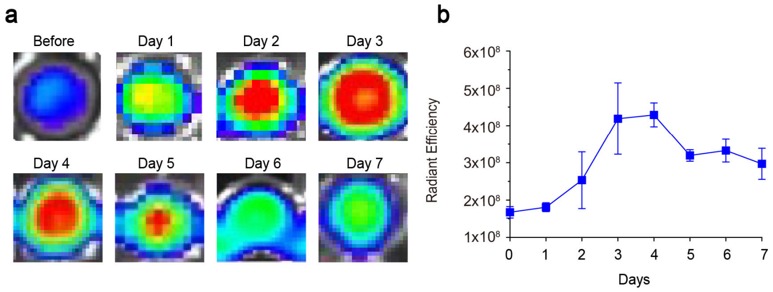

Supplementary Figure 2. Fluorescence signals of bacterium solution after ultrasound irradiation at 4.93MPa with 3s ON/ 5s OFF for 30 min. a: Fluorescence images of URB expressing mCherry protein at different time points after ultrasound irradiation. b: Quantification of fluorescence signals at different time points after ultrasound irradiation. n = 3 biologically independent samples per group. Data were presented as mean ± S.D. Source data are provided as a Source Data file.

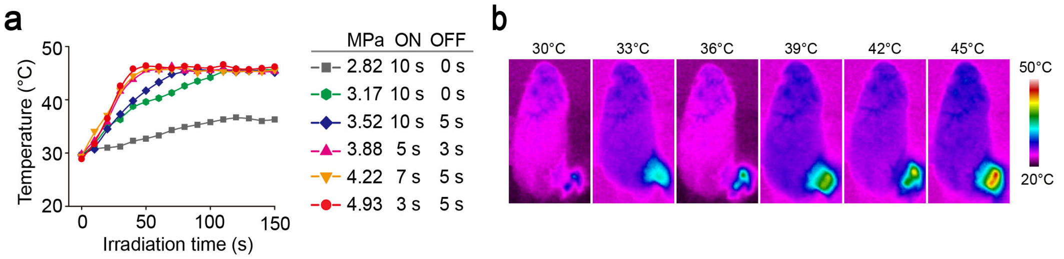

Supplementary Figure 3. Optimization of ultrasonic irradiation parameters for mice under the in vivo condition. a: Quantification of temperature changes of mouse leg irradiated by focused ultrasound at different acoustic pressures with different ON or OFF time. Gray line: 2.82 MPa, 10s ON/ 0s OFF, Green line: 3.17 MPa, 10s ON/ 0s OFF, Blue line: 3.52 MPa, 10s ON/ 5s OFF, Light red line: 3.88 MPa, 5s ON/ 3s OFF, Orange line: 4.22 MPa, 7s ON/ 5s OFF, Deep red line: 4.93 MPa, 3s ON/ 5s OFF. Experiments were performed three times independently with similar results. b: Representative infrared thermal images of the mouse legs irradiated by focused ultrasound at 3.52 MPa,10s ON/ 5s OFF. Images were representative of three experiments.

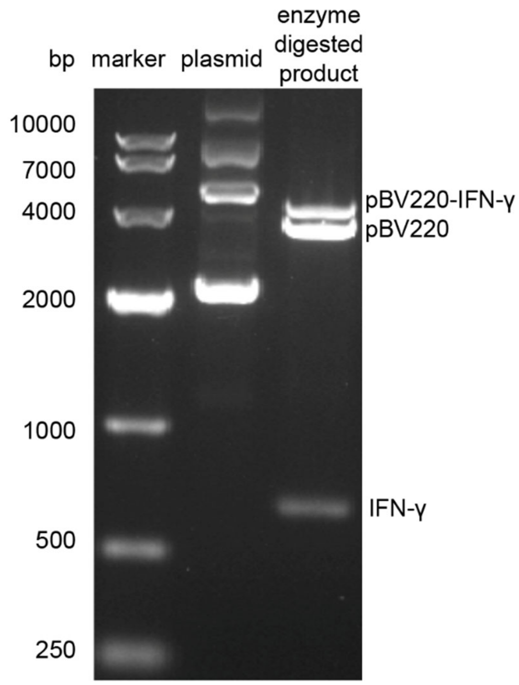

Supplementary Figure 4. Agarose gel electrophoresis of recombinant plasmid. The plasmid was cleaved by EcoRI and SalI enzymes and then performed agarose gel electrophoresis to observe the DNA band size. Panel 1 is DNA marker. Panel 2 is pBV220-IFN-γ plasmid without enzyme digestion. Panel 3 is pBV220-IFN-γ plasmid that undergone EcoRI and SalI enzyme digestion. Images were representative of three experiments. Source data are provided as a Source Data file.

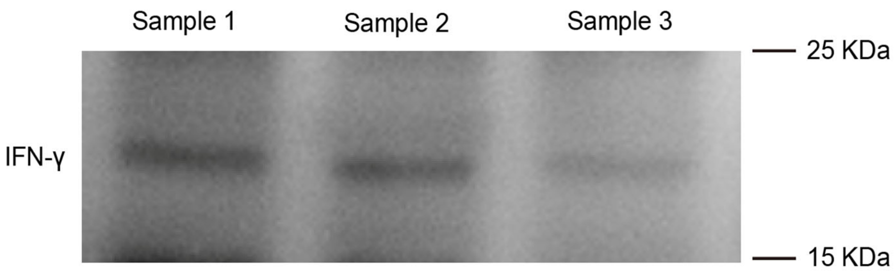

Supplementary Figure 5. Western blot analysis of the expression and secretion of IFN-γ protein. Sample 1: bacterial lysate from heating-induced URB. Sample 2: culture medium from heating-induced URB. Sample 3: bacterial lysate from URB which did not receive the heating induction. Images were representative of three experiments. Source data are provided as a Source Data file.

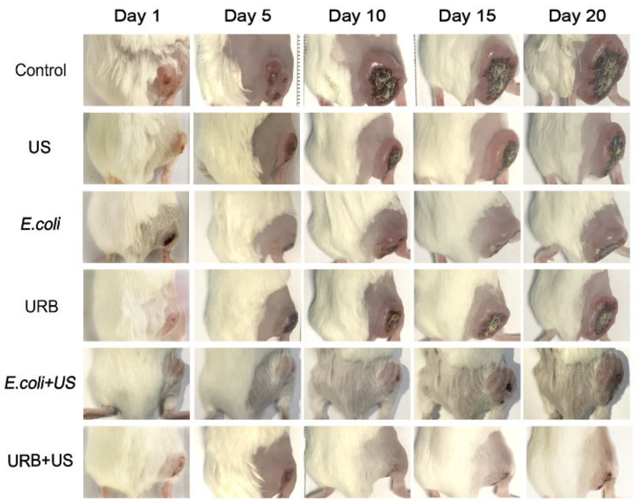

Supplementary Figure 6. Representative tumor photographs of the tumor-bearing mice after different treatments in the primary tumor inhibition experiment (corresponding to Figure 5b).

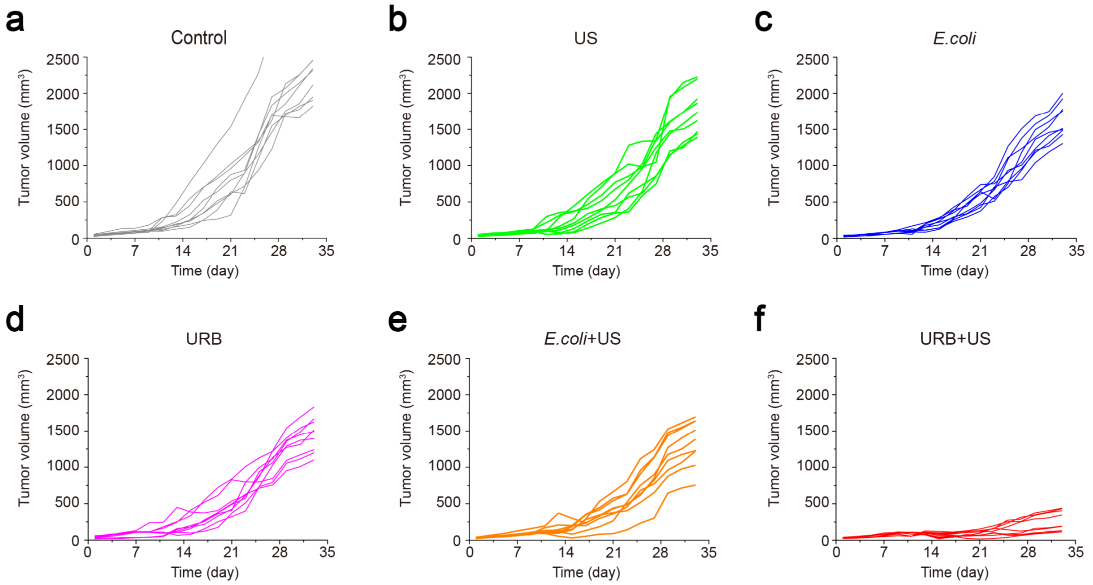

Supplementary Figure 7. Individual growth curves of 4T1 subcutaneously transplanted tumors in the primary tumor inhibition experiment (corresponding to Figure 5b). a-f: Individual tumor growth curves in different treatment group including control (a), US (b), *E.coli* (c), URB (d), *E. coli* + US (e), URB+US (f) (n = 9). Source data are provided as a Source Data file.

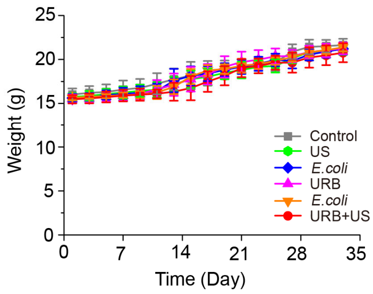

Supplementary Figure 8. Weight change of mice in different groups after treatment in the primary tumor inhibition experiment. The change curves of mouse body weight of mice bearing 4T1 tumors in different groups (corresponding to Figure 5b). n = 9 biologically independent animals per group. Data were presented as mean ± S.D. Source data are provided as a Source Data file.

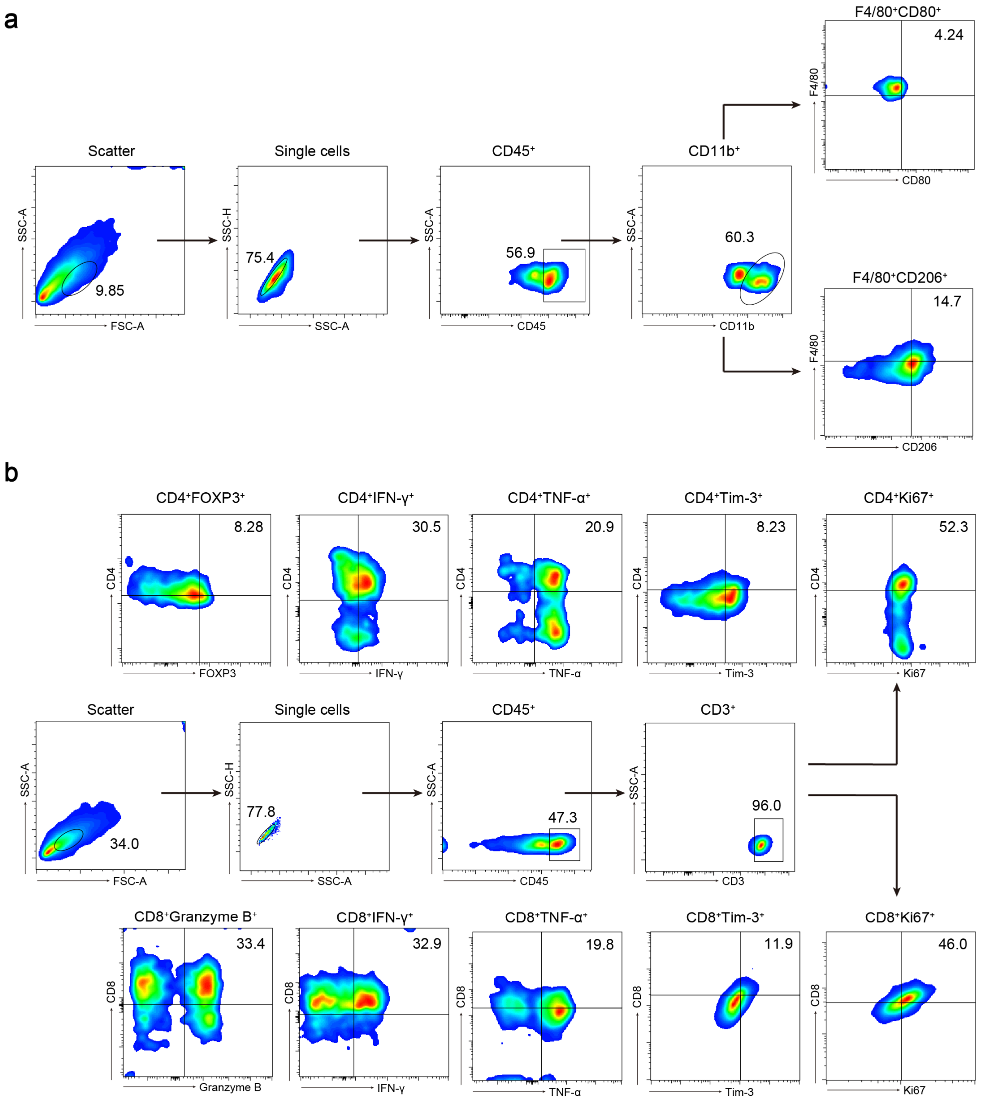

Supplementary Figure 9. Gating strategies of immune cells in the primary tumor inhibition experiment. a: Representative flow cytometry gating strategies for CD11b$^+$F4/80$^+$CD80$^+$ and CD11b$^+$F4/80$^+$CD206$^+$ macrophage panel in tumor. b: Representative flow cytometry gating strategies for CD4$^+$Ki67$^+$, CD4$^+$Tim-3$^+$, CD4$^+$TNF-α$^+$, CD4$^+$IFN-γ$^+$, CD4$^+$FOXP3$^+$, CD8$^+$Ki67$^+$, CD8$^+$Tim-3$^+$, CD8$^+$TNF-α$^+$, CD8$^+$IFN-γ$^+$, CD8$^+$GZB$^+$ T cells panel in tumor.

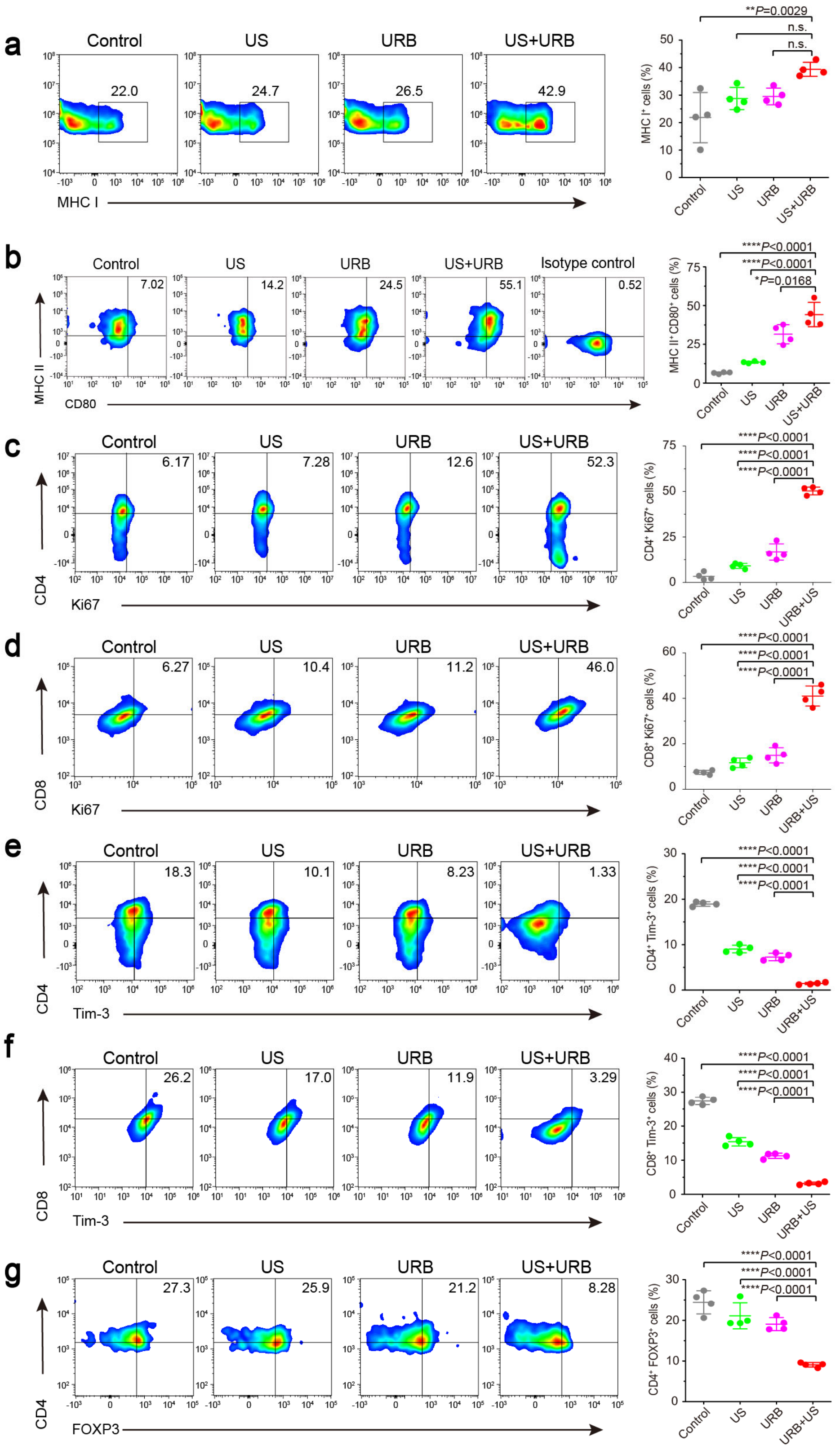

Supplementary Figure 10. Quantification of immune cells in the tumor after treatment in the primary tumor inhibition experiment. a: Flow cytometric analysis and quantification of MHC I^+ tumor cells. b: Flow cytometric analysis and quantification

of MHC $II^{hi}CD80^{+}F4/80^{+}$ cells. c: Flow cytometric analysis and quantification of $Ki67^{+}CD4^{+}$ T cells (corresponding to Figure 5j). d: Flow cytometric analysis and quantification of $Ki67^{+}CD8^{+}$ T cells (corresponding to Figure 5j). e: Flow cytometric analysis and quantification of Tim-$3^{+}CD4^{+}$ T cells (corresponding to Figure 5k). f: Flow cytometric analysis and quantification of Tim-$3^{+}CD8^{+}$ T cells (corresponding to Figure 5k). g: Flow cytometric analysis and quantification of $FOXP3^{+}CD4^{+}$ T cells (corresponding to Figure 5l). n = 4 biologically independent animals per group. Data were presented as mean ± S.D. Statistical analysis was calculated by using one-way analysis of variance with a Tukey's test (****$P < 0.0001$; ***$P < 0.001$; **$P < 0.01$; *$P < 0.05$;). Source data are provided as a Source Data file.

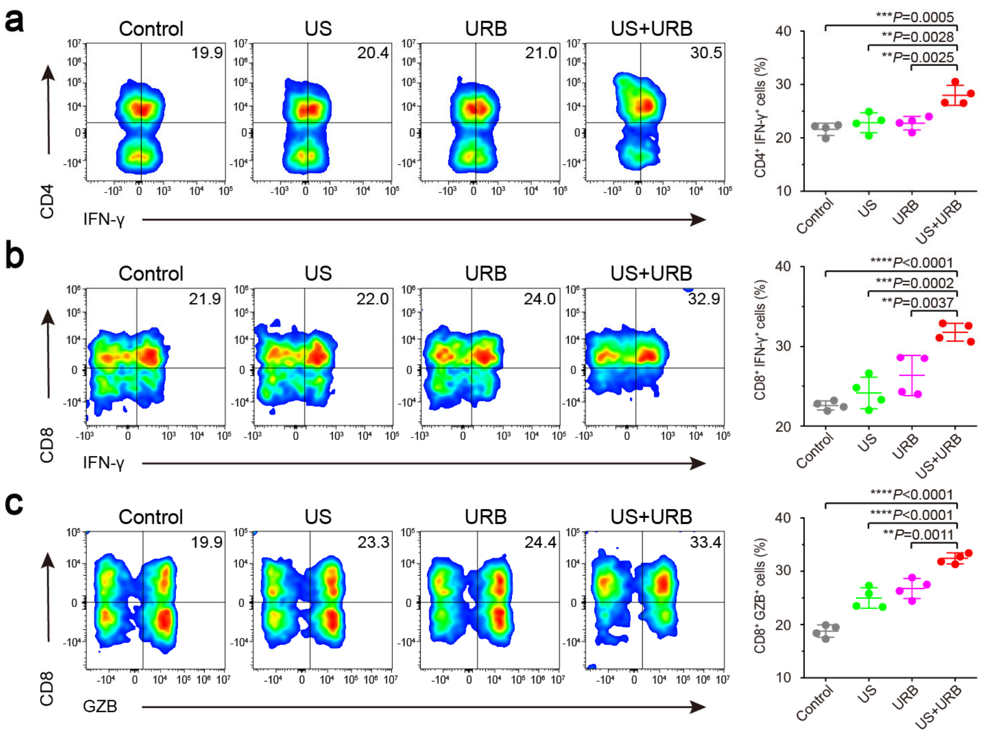

Supplementary Figure 11. Quantification of functional T cells in the tumor after treatment in the primary tumor inhibition experiment. a: Flow cytometric analysis and quantification of IFN-γ^{+}CD4^{+} T cells in tumor. b: Flow cytometric analysis and quantification of IFN-γ^{+}CD8^{+} T cells in tumor. c: Flow cytometric analysis and quantification of Granzyme B^{+}CD8^{+} T cells in tumor. n = 4 biologically independent animals per group. Data were presented as mean ± S.D. Statistical analysis was calculated by using one-way analysis of variance with a Tukey's test (*****P* < 0.0001; ****P* < 0.001; ***P* < 0.01; **P* < 0.05;). Source data are provided as a Source Data file.

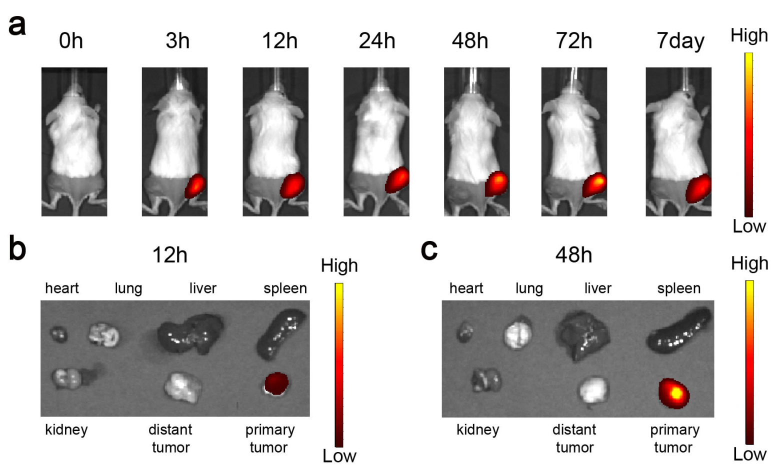

Supplementary Figure 12. The traffics kinetics of URB and gene expression between tumor and nontumor sites. a: In vivo fluorescence imaging of bilateral tumor-bearing mice at different time after US irradiation. b: The fluorescence imaging of bilateral tumors and major organs of tumor-bearing mice 12 h after US irradiation. c: The fluorescence imaging of bilateral tumors and major organs of tumor-bearing mice 48 h after intravenous injection of US irradiation. Images were representative of three experiments (n=3 animals per group).

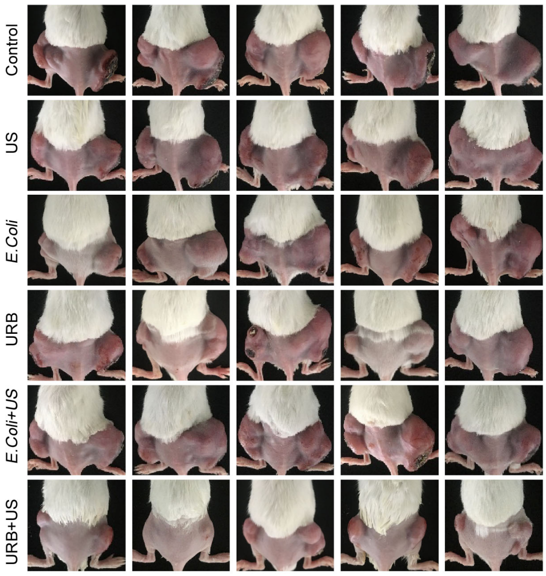

Supplementary Figure 13. Representative tumor photographs of the tumor-bearing mice after different treatments in the bilateral tumor inhibition experiment (corresponding to Figure 6b-c).

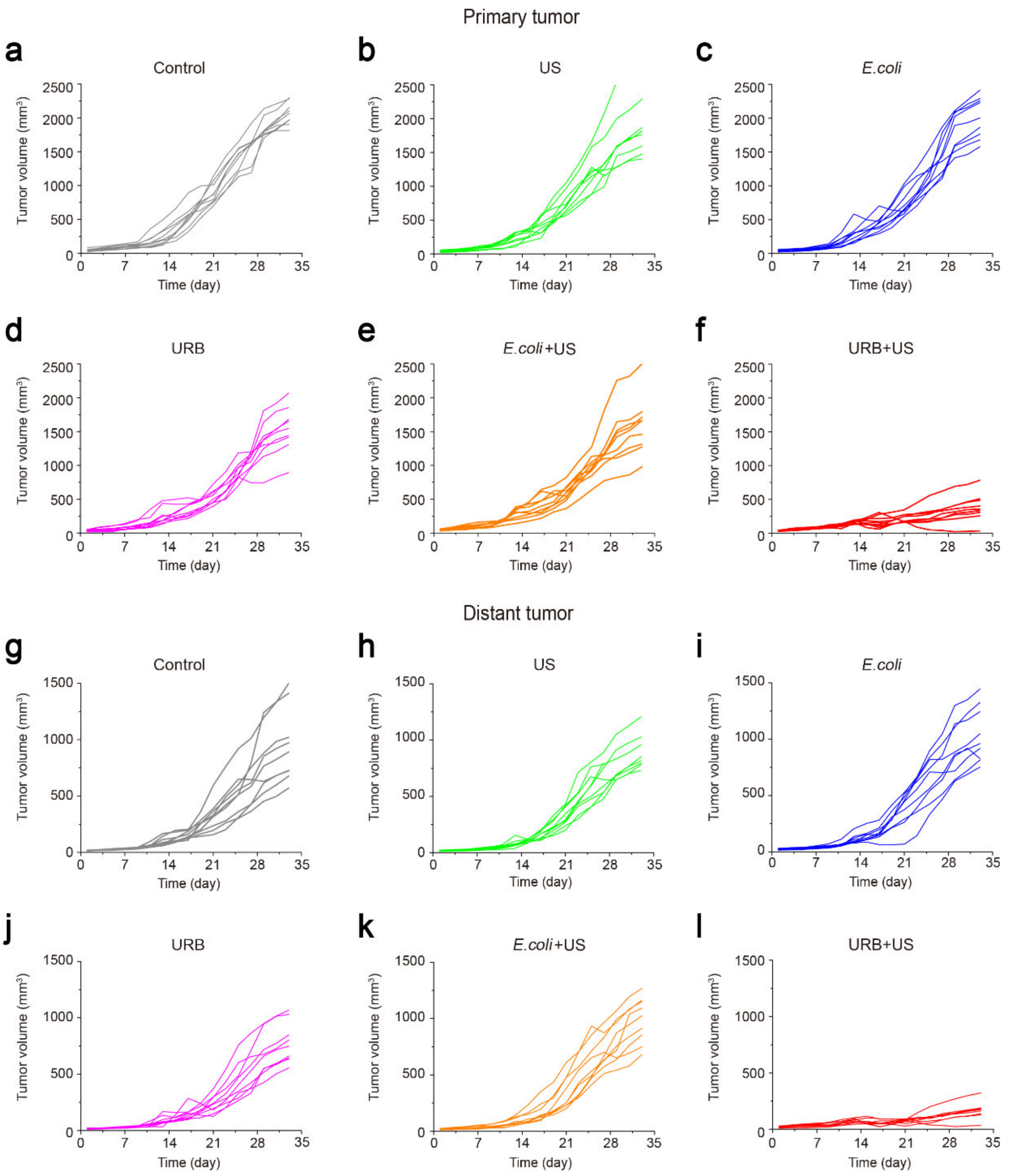

Supplementary Figure 14. Individual growth curves of 4T1 subcutaneously transplanted bilateral tumors (corresponding to Figure 6b-c). a-f: Individual growth curves of primary tumors in different treatment groups including control (a), US (b), *E.coli* (c), URB (d), *E.coli* + US (e), URB+US (f) (n = 9). g-l: Individual growth curves of distant tumors in different treatment groups including control (g), US (h), *E.coli* (i), URB (j), *E.coli* + US (k), URB + US (l) (n = 9). Source data are provided as a Source Data file.

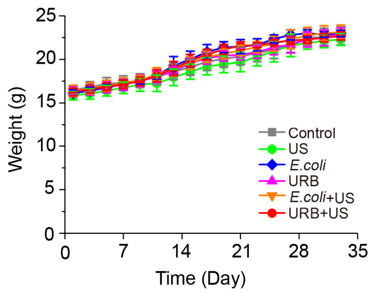

Supplementary Figure 15. Weights of mice in different groups of bilateral tumor treatment. The weights curves of mice bearing bilateral tumor in different groups (corresponding to Figure 6b-c). n = 9 biologically independent animals per group. Data were presented as mean ± S.D. Source data are provided as a Source Data file.

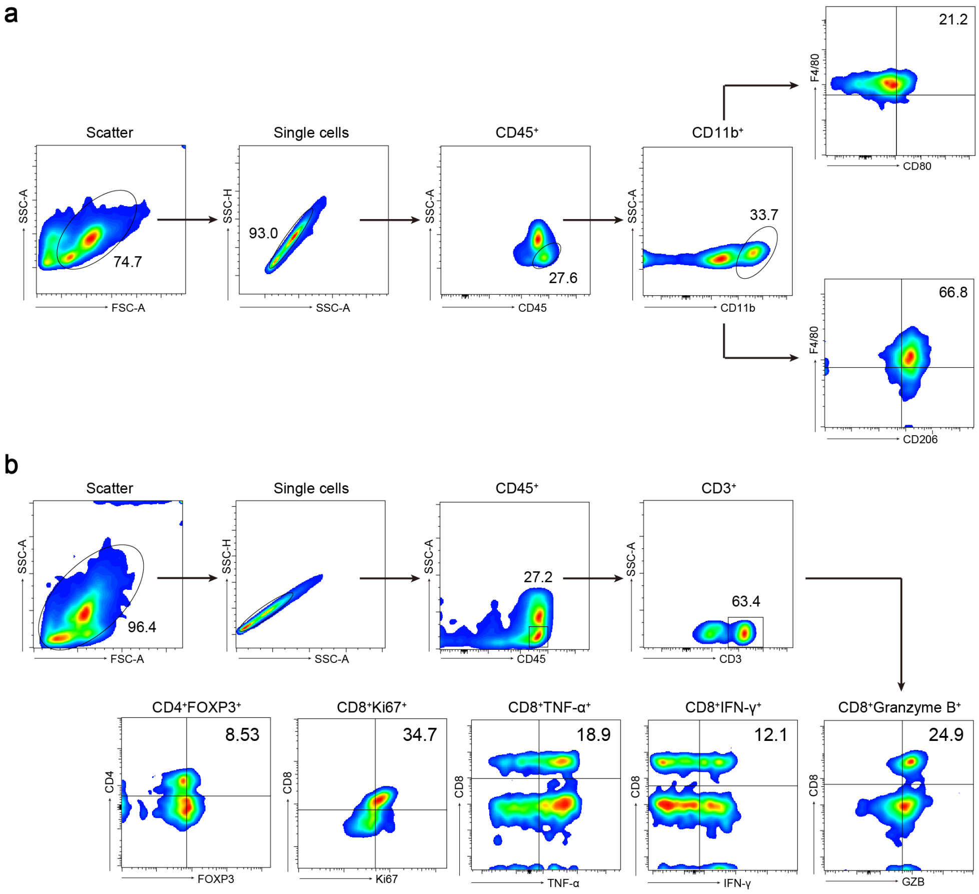

Supplementary Figure 16. Gating strategies of immune cells of spleen in the bilateral tumor inhibition experiment. a: Representative flow cytometry gating strategies for $CD11b^{+}F4/80^{+}CD80^{+}$ and $CD11b^{+}F4/80^{+}CD206^{+}$ macrophage panel in spleen. b: Representative flow cytometry gating strategies for $CD8^{+}GZB^{+}$, $CD8^{+}IFN\text{-}\gamma^{+}$, $CD8^{+}TNF\text{-}\alpha^{+}$, $CD8^{+}Ki67^{+}$ and $CD4^{+}FOXP3^{+}$ T cells panel in spleen.

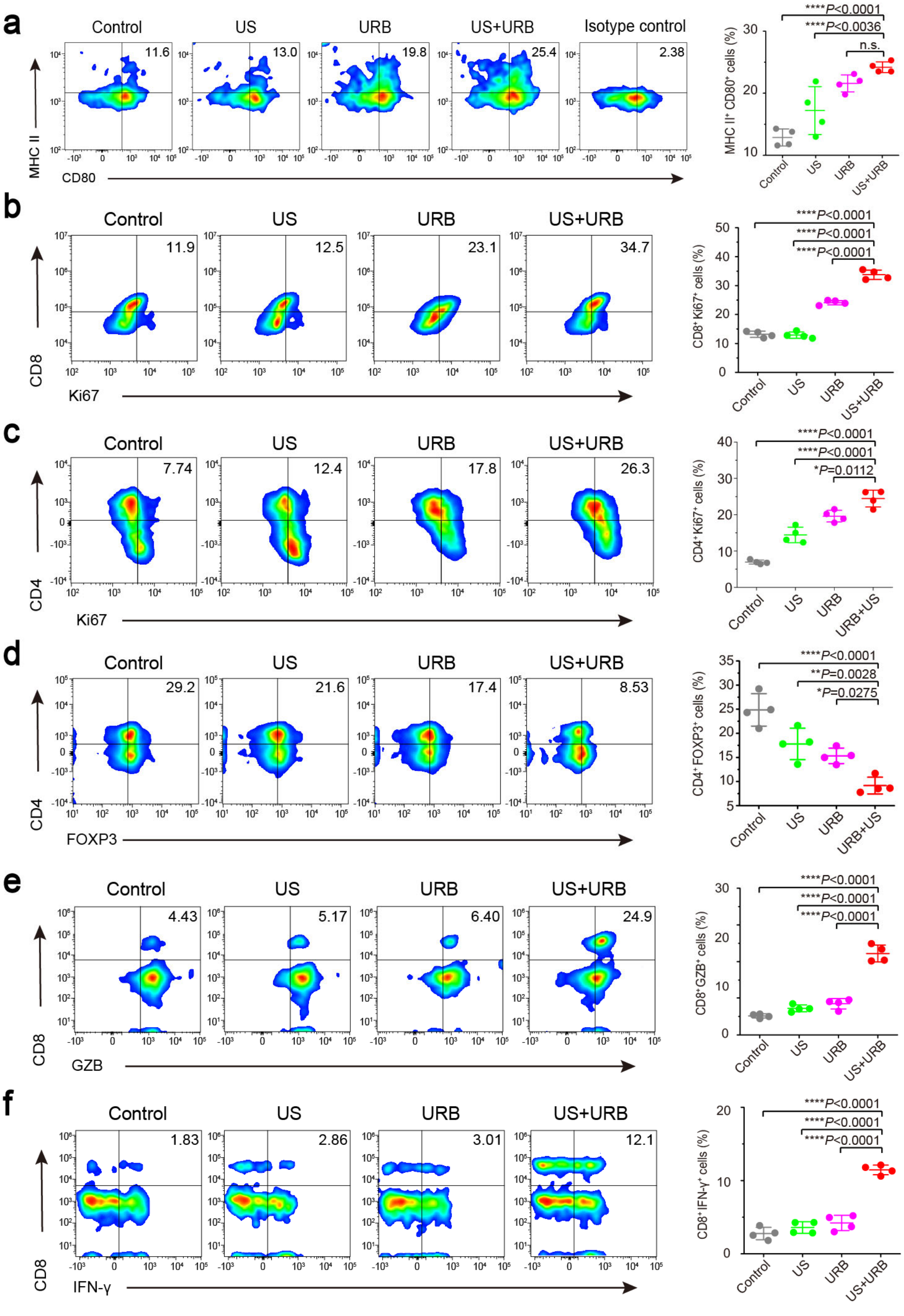

Supplementary Figure 17. Quantification of immune cells in the spleen after treatment in the bilateral tumor inhibition experiment. a: Flow cytometric analysis and quantification of MHC $II^{hi}CD80^{+}F4/80^{+}$ cells in spleen. b: Flow cytometric analysis and quantification of $Ki67^{+}CD8^{+}$ T cells in spleen (corresponding to Figure 6m). c: Flow cytometric analysis and quantification of $Ki67^{+}CD4^{+}$ T cells in spleen (corresponding to Figure 6m). d: Flow cytometric analysis and quantification of

$FOXP3^+CD4^+$ T cells in spleen (corresponding to Figure 6n). e: Flow cytometric analysis and quantification of Granzyme B^+CD8^+ T cells in spleen. f: Flow cytometric analysis and quantification of IFN-γ^+CD8^+ T cells in spleen. n = 4 biologically independent animals per group. Data were presented as mean ± S.D. Statistical analysis was calculated by using one-way analysis of variance with a Tukey's test (****P < 0.0001; ***P < 0.001; **P < 0.01; *P < 0.05;).

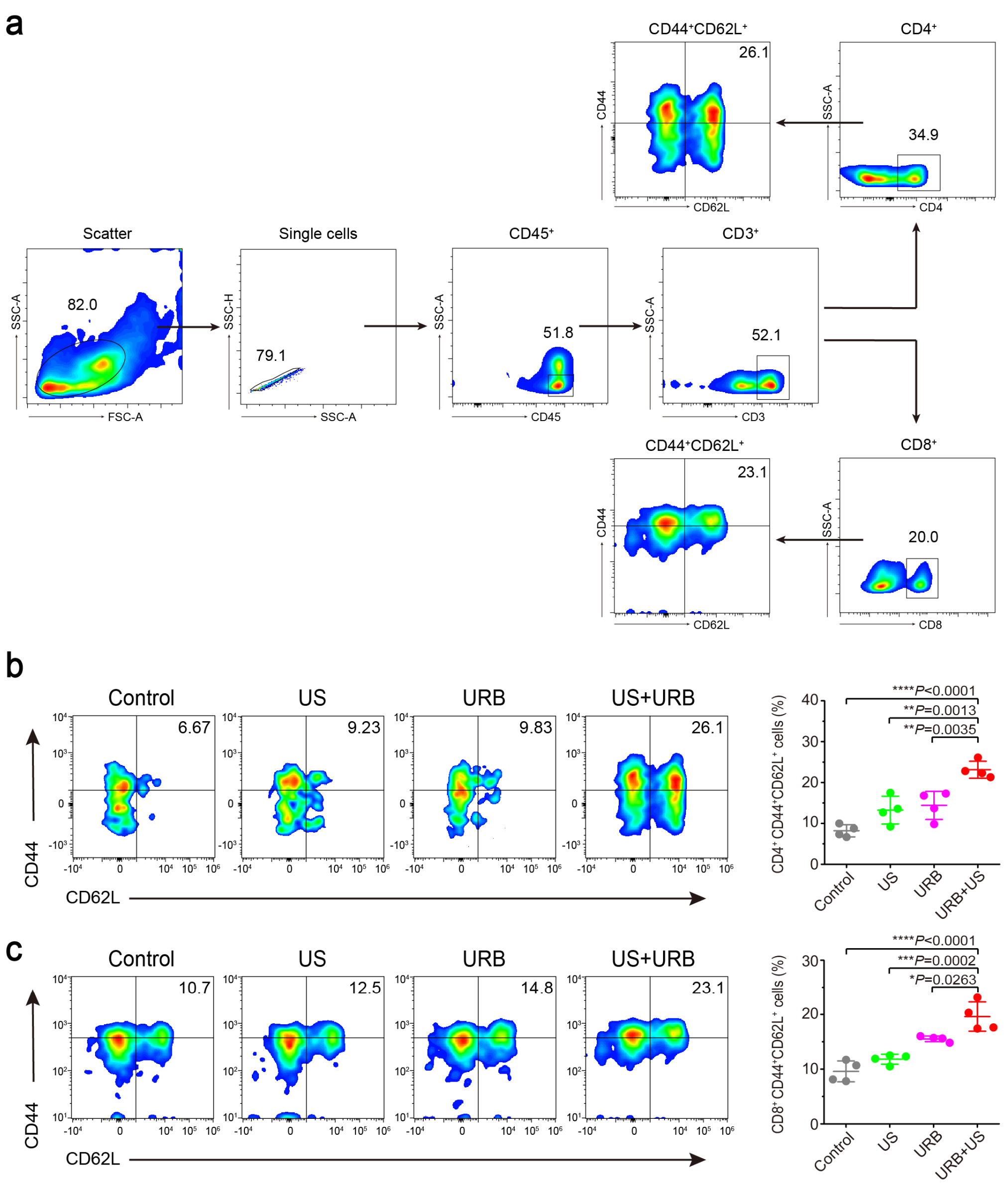

Supplementary Figure 18. Quantification of central memory T cells in the spleen after treatment in the bilateral tumor inhibition experiment. a: Gating strategies for $CD4^{+}CD44^{+}CD62L^{+}$ and $CD8^{+}CD44^{+}CD62L^{+}$ T cells panel in spleen. b: Flow cytometric analysis and quantification of $CD4^{+}CD44^{+}CD62L^{+}$ T cells in spleen(corresponding to Figure 6o). c: Flow cytometric analysis and quantification of $CD8^{+}CD44^{+}CD62L^{+}$ T cells in spleen(corresponding to Figure 6o). n = 4 biologically independent animals per group. Data were presented as mean ± S.D. Statistical analysis was calculated by using one-way analysis of variance with a Tukey's test (****P < 0.0001; ***P < 0.001; **P < 0.01; *P < 0.05;).

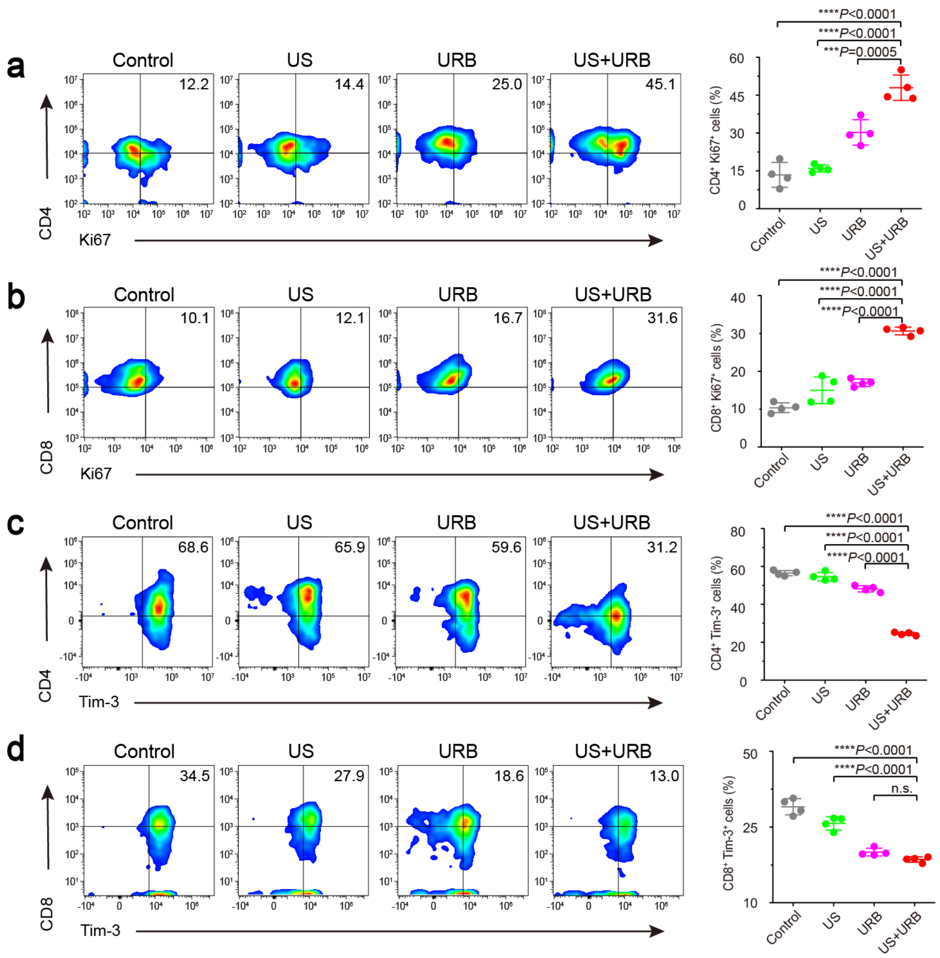

Supplementary Figure 19. Quantification of immune cells in the distant tumor after treatment in the bilateral tumor inhibition experiment. a: Flow cytometric analysis and quantification of $Ki67^+CD4^+$ T cells in distant tumor. b: Flow cytometric analysis and quantification of $Ki67^+CD8^+$ T cells in distant tumor). c: Flow cytometric analysis and quantification of Tim-3 $^+CD4^+$ T cells in distant tumor. d: Flow cytometric analysis and quantification of Tim-3 $^+CD8^+$ T cells in distant tumor. Data were presented as mean ± S.D. Statistical analysis was calculated by using one-way analysis of variance with a Tukey's test (****$P < 0.0001$; ***$P < 0.001$; **$P < 0.01$; *$P < 0.05$;). Source data are provided as a Source Data file.

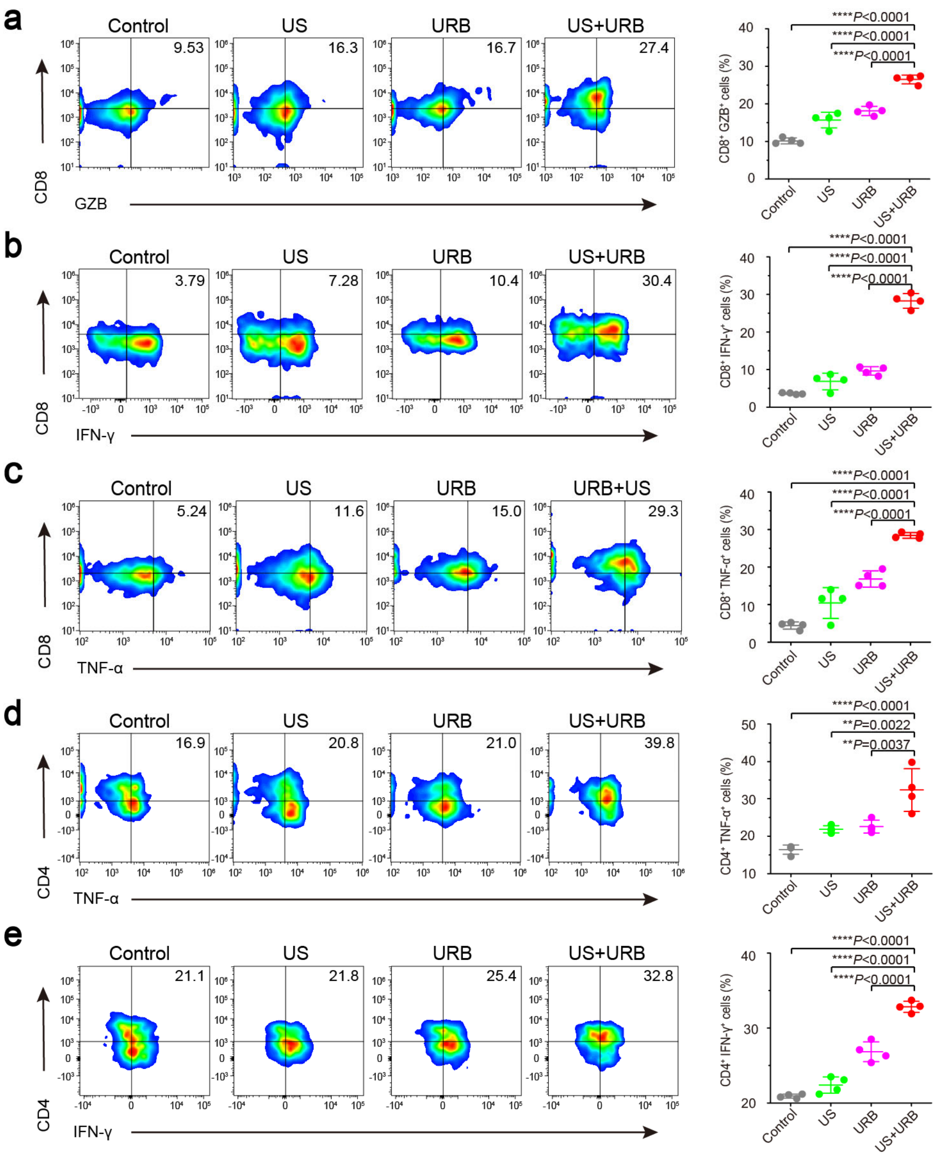

Supplementary Figure 20. Quantification of immune cells in the distant tumor after treatment in the bilateral tumor inhibition experiment. a: Flow cytometric analysis and quantification of Granzyme B^+CD8^+ T cells in distant tumor. b: Flow cytometric analysis and quantification of IFN-γ^+CD8^+ T cells in distant tumor. c: Flow cytometric analysis and quantification of TNF-α^+CD8^+ T cells in distant tumor. d: Flow cytometric analysis and quantification of TNF-α^+CD4^+ T cells in distant tumor. e: Flow cytometric analysis and quantification of IFN-γ^+CD4^+ T cells in distant tumor. n = 4 biologically independent animals per group. Data were presented as mean ± S.D. Statistical analysis was calculated by using one-way analysis of variance with a Tukey's test (****P < 0.0001;***P < 0.001; **P < 0.01; *P < 0.05;). Source data are provided as a Source Data file.

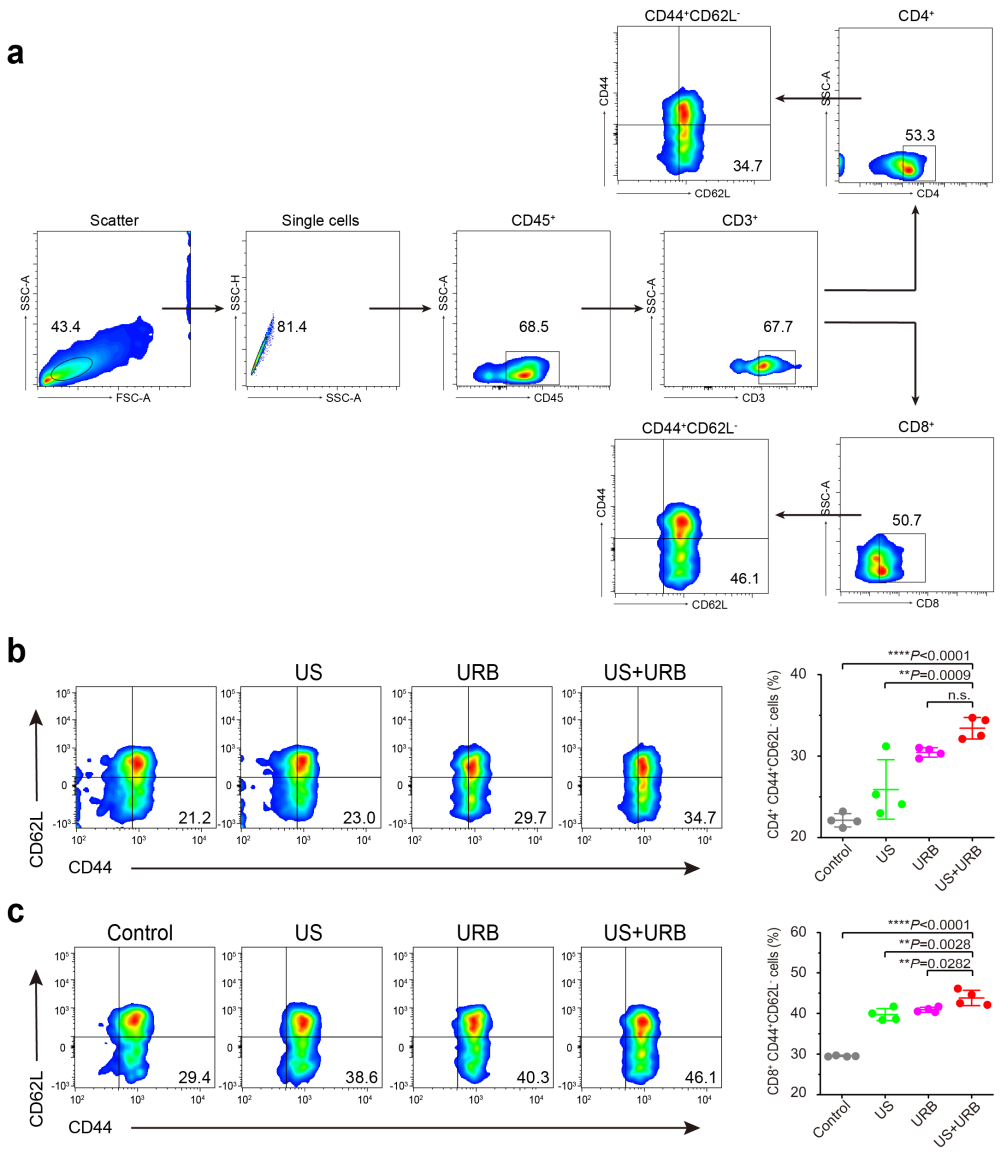

Supplementary Figure 21. Quantification of effector memory T cells in distant tumor after treatment in the bilateral tumor inhibition experiment. a: Gating strategies for $CD4^+CD44^+CD62L^-$ and $CD8^+CD44^+CD62L^-$ T cells panel in distant tumor. b: Flow cytometric analysis and quantification of $CD4^+CD44^+CD62L^-$ T cells in distant tumor(corresponding to Figure 6t). c: Flow cytometric analysis and quantification of $CD8^+CD44^+CD62L^-$ T cells in distant tumor(corresponding to Figure 6t). n = 4 biologically independent animals per group. Data were presented as mean ± S.D. Statistical analysis was calculated by using one-way analysis of variance with a Tukey's test (****$P < 0.0001$; ***$P < 0.001$; **$P < 0.01$; *$P < 0.05$;). Source data are provided as a Source Data file.

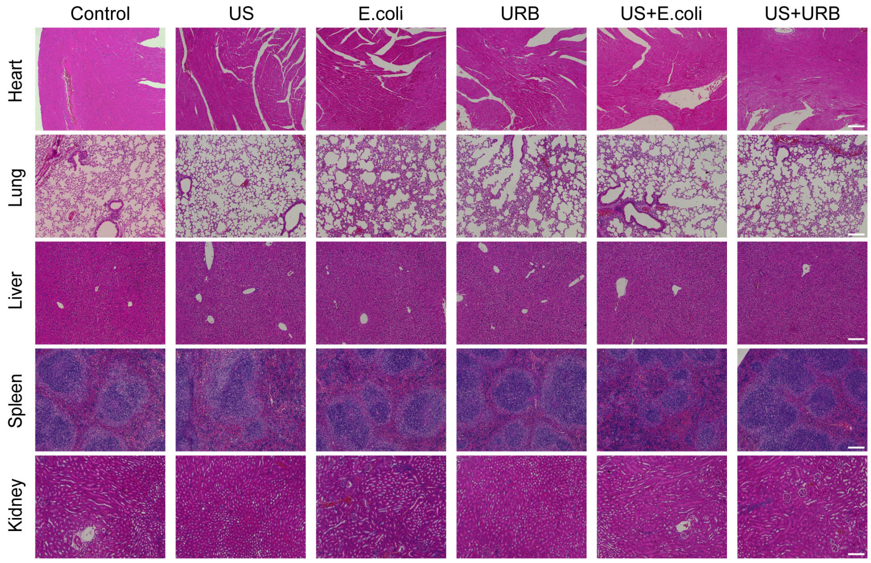

Supplementary Figure 22. Representative images of H&E staining organ slices of different group mice with different treatment. Scale bar = 100 μm. Images were representative of three experiments (n=3 animals per group).

Supplementary tables

Supplementary table 1. Blood biochemistry of mice from the *in vivo* treatment experiment.

	Control	US	*E.coli*	URB	US+ *E.coli*	US+URB	Normal ranges
Alanine aminotransfe rase (ALT) (U/L)	82.75±9.67	70.03±12.36	74.63±8.14	83.22±6.34	84.75±7.93	69.97±8.18	10.06-96.47
Aspartate aminotransfe rase (AST) (U/L)	112.71±11.96	100.13±7.61	107.72±3.74	98.88±5.32	102.10±5.91	116.31±17.16	36.31-235.48
Uric nitrogen (BUN) (mg/dL)	20.37±1.40	19.99±0.83	22.69±0.83	18.97±1.72	20.65±0.90	21.68±1.13	10.81-34.74
Creatinine （Cr）(umol/L)	20.13±0.25	20.14±0.88	23.59±0.86**	20.27±0.68	22.55±0.96	24.01±1.60**	10.91-85.09
Direct Bilirubin (DBIL) (umol/L)	10.61±0.56	10.91±0.66	12.54±1.37	10.98±0.49	12.73±0.68	13.64±0.60**	0.45-33.89
Total bilirubin (TBIL) (umol/L)	18.09±1.13	16.49±1.36	19.15±0.84	20.45±0.69	19.66±0.71	20.39±1.21	6.09-53.06

Note: n = 3 biologically independent animals samples per group. Data were presented as mean ± S.D. Statistical analysis was calculated by using one-way analysis of variance with a Tukey's test (Creatinine: **P = 0.0084 *E.coli* vs control group; **P = 0.0035 US+URB vs control group. Direct Bilirubin: **P = 0.0050 US+URB vs control group). Source data are provided as a Source Data file.

Supplementary table 2. Blood routine of mice from the *in vivo* treatment experiment.

	Control	US	E.coli	URB	US+E.coli	US+URB	Normal ranges
WBC (10^9/L)	3.54±0.47	4.17±0.98	3.72±0.45	3.87±0.32	3.61±0.25	3.83±0.50	0.8-6.8
Lymph# (10^9/L)	2.70±0.20	3.43±0.61	2.67±0.21	2.80±0.10	2.50±0.10	3.00±0.17	0.7-5.7
Mon# (10^9/L)	0.17±0.06	0.23±0.06	0.23±0.06	0.13±0.06	0.13±0.06	0.23±0.06	0.0-0.3
Gran# (10^9/L)	0.93±0.06	1.00±0.20	1.13±0.21	0.83±0.15	1.13±0.21	1.13±0.12	0.1-1.8
Lymph% (%)	65.73±1.17	74.10±4.44	69.03±2.18	73.43±5.45	65.30±2.36	72.90±4.73	55.8-90.6
Mon% (%)	5.47±0.40	4.60±0.30*	4.57±0.21*	4.27±0.38**	3.87±0.15***	4.87±0.06	1.8-6.0
Gran% (%)	31.07±0.72	22.47±3.40**	29.40±1.31	21.47±3.96**	30.33±1.91	29.50±1.10	8.6-38.9
RBC (10^{12}/L)	8.47±0.43	7.87±0.09	7.93±0.15	7.37±0.45*	7.63±0.39	8.00±0.13	6.36-9.42
HGB (g/L)	126.3±13.58	111.7±6.66	111.3±2.52	116.0±2.64	110.7±3.21	119.7±7.51	110-143
HCT (%)	39.6±0.92	36.5±1.81	37.5±1.05	35.5±2.57	37.3±1.10	37.3±2.75	34.6-44.6
MCV (fl)	46.7±0.40	46.6±0.21	46.6±0.26	47.1±1.47	46.7±0.47	46.7±0.50	48.2-58.3
MCH (pg)	14.5±0.29	14.7±0.10	14.5±0.36	14.8±0.17	14.6±0.10	14.6±0.25	13.7-16
MCHC (g/L)	316.7±2.08	316.0±1.73	312.3±2.52	305.0±7.21	313.0±5.57	318.3±4.51	302-353
RDW (%)	17.3±0.75	19.6±0.73*	17.6±0.52	18.6±0.76	18.3±0.99	17.7±0.38	15.05-20.85
PLT (10^9/L)	709.7±91.0	632.3±6.66	590.7±28.4	399.0±55.9***	504.7±20.8**	511.7±6.43**	450-1590
MPV (fl)	5.60±0.26	5.60±0.10	5.83±0.23	5.33±0.45	5.97±0.21	5.73±0.15	3.8-6.0

Note: n = 3 biologically independent animals samples per group. Data were presented as mean ± S.D. Statistical analysis was calculated by using one-way analysis of variance with a Tukey's test (Mon%: **P* = 0.0235 US vs control group; **P* = 0.0184 *E.coli* vs control group; **P* = 0.0021 URB vs control group; ****P* <0.001 US+*E.coli* vs control group. Gran%: ***P* = 0.0086 US vs control group; ***P* = 0.0037 URB vs control group. RBC: **P* = 0.0100 URB vs control group. RDW: **P* = 0.0189 US vs control group. PLT: ****P* <0.001 URB vs control group; ***P* =0.0016 US+*E.coli* vs control group; ***P* =0.0021 US+URB vs control group). Source data are provided as a Source Data file.
